# Biological, dietetic and pharmacological properties of vitamin B_9_

**DOI:** 10.1038/s41538-025-00396-w

**Published:** 2025-03-13

**Authors:** Tomáš Siatka, Marek Mát’uš, Monika Moravcová, Patrícia Harčárová, Zuzana Lomozová, Kateřina Matoušová, Chaweewan Suwanvecho, Lenka Kujovská Krčmová, Přemysl Mladěnka

**Affiliations:** 1 Department of Pharmacognosy and Pharmaceutical Botany, Faculty of Pharmacy in Hradec Králové, Charles University, Akademika Heyrovského 1203, 500 03 Hradec Králové, Czech Republic; 2 Department of Pharmacology and Toxicology, Faculty of Pharmacy, Comenius University Bratislava, Odbojárov 10, 83232 Bratislava, Slovak Republic; 3 Department of Pharmacology and Toxicology, Faculty of Pharmacy in Hradec Králové, Charles University, Akademika Heyrovského 1203, 500 03 Hradec Králové, Czech Republic; 4 Department of Clinical Biochemistry and Diagnostics, University Hospital Hradec Králové, Sokolská 581, 500 05 Hradec Králové, Czech Republic; 5 Department of Analytical Chemistry, Faculty of Pharmacy in Hradec Králové, Charles University, Akademika Heyrovského 1203, 500 03 Hradec Králové, Czech Republic

**Keywords:** Pathogenesis, Biochemistry

## Abstract

Humans must obtain vitamin B_9_ (folate) from plant-based diet. The sources as well as the effect of food processing are discussed in detail. Industrial production, fortification and biofortification, kinetics, and physiological role in humans are described. As folate deficiency leads to several pathological states, current opinions toward prevention through fortification are discussed. Claimed risks of increased folate intake are mentioned as well as analytical ways for measurement of folate.

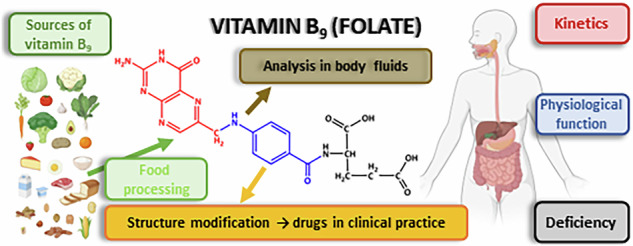

## Introduction

Vitamin B_9_ is one of the crucial vitamins whose physiological function and consequence of its deficiency are well known, however, there is a lack of a comprehensive paper summarizing essential aspects of this vitamin encompassing its natural occurrence, the impact of different factors on its stability and absorption as well as its further fate in the human organism in the context of its possible deficiency with its causes, and consequences, as well as current discussion on possible risks of high dose folate supplementation. This paper aims to offer such a complex review.

**Fig. 1:**
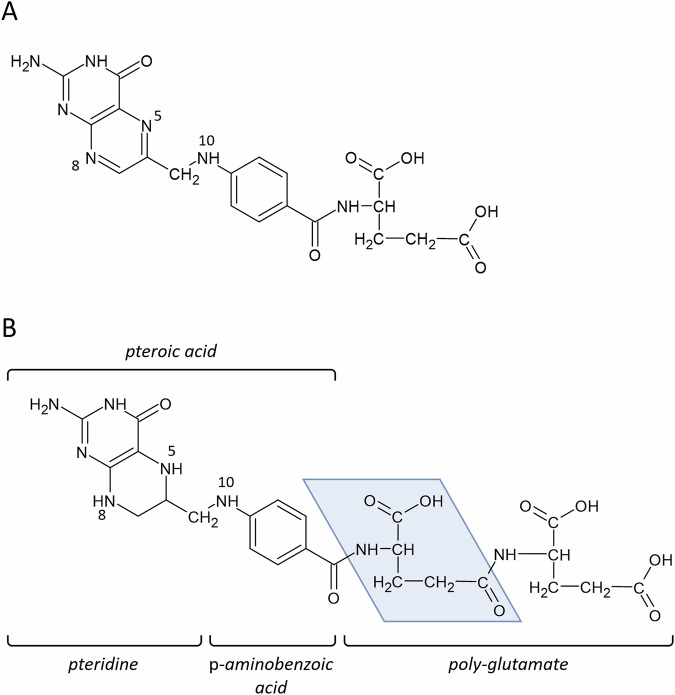
General folate structure. **A** Folic acid; **B** tetrahydrofolate. The blue area marks a monoglutamate unit of the polyglutamate chain.

Vitamin B_9_, or folate, is a generic term given to a group of chemically related molecules based on the folic acid structure (Fig. 1). These molecules contain a pteridine heterocycle that can be in a reduced or oxidized form; a *p*-aminobenzoic acid bridge and a mono-/polyglutamate chain of variable length. Additionally, one carbon unit can be bound to either the pteridine ring, *p*-aminobenzoic moiety, or both. Folic acid is the most oxidized folate form. Folic acid can be reduced at nitrogen-8 to produce dihydrofolate. Further reduction at nitrogen-5 generates the active coenzyme form: tetrahydrofolate (THF). Both reductive steps are catalysed by the enzyme dihydrofolate reductase.

Reduced tetrahydrofolate may serve as an acceptor of one-carbon units via nitrogen-5 and nitrogen-10. These carbon units can bind in different oxidation states and generate different forms of tetrahydrofolate cofactors which have distinct physiological functions: 5-methyl-THF; 5,10-methylene-THF (methylene-THF) and 10-formyl-THF. A synthetic folate molecule, 5-formyl-THF (folinic acid), is often used in medications.

## Sources of vitamin B_9_

### Folate – vitamin B_9_

Plants, fungi, certain protozoa, several archaea, and many bacteria can synthesize folate de novo. Animals and humans are unable to synthesize folate and entirely depend on an adequate and constant intake of the vitamin from exogenous sources^1–32^. Folate occurs in a wide variety of foods. Dark green vegetables (e.g., spinach, broccoli, Brussels sprouts, and romaine lettuce), cereals (especially whole grains), fruits (e.g., oranges, papaya, and avocado), and legumes (e.g., chickpea, soybean, and lentil) are the major sources^1,13,33–89^. Liver, yeast, and egg yolk contain also very high amounts of folate^1,33,50,60,75,90–105^. Logically, the contribution of every dietary source to meet the daily folate requirements depends on its consumption in the general population. For example, yeast, liver, and pulses, which are rich in folate, contribute less to folate supply due to their low consumption, in contrast to vegetables and fruits with lower folate contents but higher consumption^106–123^. In the United Kingdom, Ireland, and Sweden, bread as a typical cereal product provides, on average, 9–14%, 14%, and 13%, resp., of dietary folate daily^124–128^. Similarly, bread and rolls contribute to the folate intake in the average Polish diet by 17%^129^. Pseudocereals such as quinoa and amaranth are suitable alternatives to gluten-containing cereals (wheat, barley, and rye), in terms of folate content, for people with coeliac disease^88,130–134^. Meat and meat products, except for the liver, which is the folate storage organ in mammals, contain little folate^75,135^. Folate status tends to be higher in people eating plant-based diets as compared to meat-eaters (i.e., omnivores), with the highest levels of folate being in vegans^115,136–145^. Milk and fermented dairy products, which are not considered rich in folate, constitute an important dietary source of folate because they are consumed in relatively large quantities; milk is responsible for 10–15% of the daily folate intake in European countries. This is particularly important for high milk and dairy products consuming countries such as the Netherlands, Sweden, and Spain^1,34,50,98,107,115,117,146–149^. Potatoes do not have high folate content but are also the major source of the vitamin due to the high frequency of their consumption; potatoes supply about 10% of the total folate intake of people in European countries such as the Netherlands, Ireland, Norway, and Finland^67,98,115,117,128,141,148,150,151^. Younger potato tubers (‘new potatoes’) are richer in folate than mature ones; higher consumption of new or baby potatoes could significantly increase folate intake^152,153^. Some wild vegetables and fruits contain amounts of folate comparable to those in conventional ones and may serve for the diversification of our current diet to increase folate intake^154–157^. Similarly, some microalgae, e.g., *Chlorella*, but not *Arthrospira* (*Spirulina*), seaweeds, and yeasts, such as *Yarrowia lipolytica*, could represent an additional source of folate in the human diet^109,119,158–166^. Several mushrooms that are higher in folate (e.g., oyster and enoki) could enhance natural folate intake as well^109,162,167–170^. Edible insects, such as mealworms and crickets, may also enrich the human diet with folate^171–173^.

Data on the folate content in foods vary. Variations, especially in foods of plant origin, could be attributed to factors such as plant varieties and cultivars, growing conditions (e.g., season and climate), and agronomic practices (e.g., harvest time and postharvest handling). A microbiological assay is a widely accepted official method for folate measurement in many countries. Differences in the analytical methodology may also affect the measured folate content^41,56,98,106,174–183^. The contents of folate in some selected foodstuffs are shown in Table 1.

**Table 1:** Folate contents in selected foodstuffs

Food	Folate content (μg/100 g)	References
Oat	29–69	^34,105,327,375,618,770,813,1227^
Wheat	38–74	^124,319,375,759,760,813^
Rice, brown	20–41	^34,319,328–330,375,376,443,579,618,1228^
Rice, white	6–14	^34,319,328–330,375,376,579,618,1228^
Maize	19–31	^319,323,375,618,1227^
Rye	38–78	^34,319,618,761,768,769,813,1227,1229^
Barley	23–79	^162,163,304,319,324,618,761,767,813,1227^
Millet	36–85	^618,813,1230^
Sorghum	20–55	^34,319,1227,1230,1231^
Soybean	260–375	^163,375,415,443,618^
Lentil	147–479	^106,618,1227^
Peanut	95–240	^105,163,376,1232–1234^
Macadamia nut	11	^618,1235^
Pistachio nut	44–93	^41,618,1227,1235^
Hazelnut	90–113	^329,618,1235^
Walnut	73–98	^329,1227,1234,1235^
Almond	44–65	^329,375,618,1234–1236^
Garlic	3–20	^329,376,618^
Potato	13–27	^75,102,162,163,375–377,618,1227,1237^
Carrot	8–31	^13,60,75,162,163,375–377,618,1237^
Cabbage	43–84	^75,106,162,163,329,375–377,618,1227^
Tomato	15–33	^60,75,105,163,177,181,329,375,377,486,618,1227,1238^
Broccoli	63–155	^75,102,105,376,377,618,1227,1237,1239^
Cauliflower	55–67	^13,75,177,329,376,377^
Spinach	194–264	^75,102,106,162,163,177,182,375,376,618,1227,1237,1239,1240^
Orange	30–46	^34,163,177,329,376,377,618,1227,1238^
Avocado	31–89	^34,75,376,618,1239,1241^
Strawberry	24–96	^34,74,75,105,106,329,375,377,1227,1238^
Apple	3–6	^61,105,106,163,329,375,377,618,1227,1238,1239^
Pear	4–7	^61,375–377,1227,1238,1242^
White bread	24–39	^75,102,125,163,177,325,375,376,1227,1243^
Brown bread	27–45	^125,177,325,376,1243^
Pork	2–6	^105,135,162,163,375,376^
Beef	4–11	^135,162,163,375,376^
Chicken breast	4–13	^105,162,163,329,375,1244^
Liver, beef	290–1000	^375,376,1239,1242^
Liver, pork	212–810	^105,375,376,1242^
Tuna	4–6	^375,378^
Sardines	7–10	^375,376,378^
Baker’s yeasts	785–1250	^75,376,1242^
Oyster mushroom	38–92	^163,170,375,1242^
Button mushroom	19–46	^170,329,375,1242,1245^
Milk	5–8	^53,105,149,163,375,461,618,1227,1239,1246,1247^
Youghurt	11–18	^34,53,105,149,329,375,376,1227,1246,1247^
Cheese, cheddar	19–32	^149,329,375,376,1242,1247^
Eggs	43–74	^34,100,163,329,375,376,1227^

Based on several human studies, food folate (a mixture of natural reduced pteroylmono- and polyglutamates) has a lower bioavailability than synthetic monoglutamate folic acid added to foods for supplementation and food fortification purposes. Folic acid is absorbed almost completely when taken without simultaneous consumption of food, whereas its bioavailability from fortified foods or supplements ingested during a meal is about 85%. The bioavailability of food folate is estimated to be around 50%, i.e., half that of folic acid taken with water on an empty stomach, due to losses during digestion and absorption. In general, folic acid in fortified products or taken with foods is 85/50 or 1.7 times more bioavailable than food folate. Several factors may hinder the absorption of natural food folate, e.g., partial release from the food matrix (incomplete liberation from cellular structures), destruction within the gastrointestinal tract, and incomplete hydrolysis of polyglutamates to monoglutamates (possibly mediated by partial inhibition of deconjugation enzymes by other dietary constituents such as organic acids). On the contrary, such factors are negligible in the case of added folic acid, which does not require the release from cellular structures, is more stable and less susceptible to destruction within the lumen than natural food folate, and exists as a monoglutamate, i.e. the form necessary for normal absorption in the small intestine (see Absorption section below). The bioavailability of supplemental 5-methyl-THF has been reported to be similar or higher compared to folic acid at equimolar doses. A typical diet would contain a combination of food folate and folic acid provided in fortified products or supplements; the dietary folate (‘dietary folate equivalents’) would then be computed as follows: μg food folate + (1.7 x μg folic acid). Although there is a broad agreement that naturally occurring food folate is not as bioavailable as folic acid, uncertainties still exist in relation to the extent of these differences, particularly in the context of a whole diet. Some studies indicate that the bioavailability of food folate is underestimated and is higher than the generally assumed value of 50%. Therefore, more research is needed for a better understanding of folate bioavailability from food and influencing factors^1,37,75,98,120,146,147,149,184–247^.

There is a paucity of data on the possible contribution of folate, which is produced by microorganisms in the colon, to the overall human body’s needs for folate. It might be a complementary endogenous source of folate to that derived from the diet^248,249^. A part of the microbiota in the human large intestine is capable of synthesizing folate (folate prototrophs); the rest microbiome members lacking the ability, however, are consumers (folate auxotrophs) and rely on those folate producers to provide folate, which may limit the vitamin availability for the host including humans^248,250–259^. It is known that the human gut microbiome is different and stratified, not continuous, in the population. It may be clustered into three enterotypes according to the species composition and functional properties. Although all vitamin metabolic pathways were represented in all microbiome samples, enterotypes 1 and 2 are enriched in genes involved in the biosynthesis of different vitamins, those for thiamine and folate being in enterotype 2. It may be beneficial to the human host^260,261^. The abundance of folate biosynthetic genes in human colon microbiome may change with the age^262–264^. Moreover, microbial folate production in the colon may be influenced by diet. Intake of soluble, fermentable dietary fibres enhanced plasma folate concentrations in rats and humans, bacterial load, and total folate content in the colon, but not the whole body’s folate status in piglets^265–267^. A positive effect of supplementation with folate-producing bifidobacteria on folate plasma levels has been observed in a rat experiment as well as in a human trial^268,269^, but not in a mouse experiment^270^. In another rat experiment, it was found that folate derived from caecal bacteria is not absorbed and does not increase the liver folate stores^271^. It has been shown that microbially synthesized folate can be partly absorbed across the large intestine in piglets^272^. Absorption of isotopically labelled 5-formyl-THF across the colon at a considerably lower rate (about one-fiftieth) than across the small intestine has been reported. However, the difference in the net absorption was estimated to be smaller (approximately one-tenth) due to much longer transit in the colon than in the small intestine^273–275^. The existence of a folate transporter in the human colonic cells has been demonstrated; it is expressed at much lower levels in the cells in the colon than in the small intestine, where folate absorption primarily occurs^276^. Thus, in situ produced microbial folate may favourably influence the cellular nutrition of the local colonocytes and may be important in maintaining intestinal homeostasis and modulating gut microbiome function, e.g., through regulation of colon mucosal proliferation (i.e., colorectal cancer prevention) and its anti-inflammatory effects^248,249,256,257,277–286^. However, there are still a lot of questions that remain to be answered about the relationship between folate levels in the colonic mucosa and the systemic circulation and the colorectal cancer risk, and about the role of folate derived from the diet and that from local microbial production^287–290^. Regardless, it is still unknown whether folate synthesized by the human colonic microbiota can substantially affect the body’s general folate status as this has never been sufficiently quantified to date^90,211,233,248,251,277,286,291^.

#### Impact of food processing and storage on folate contents

Food processing and storage can greatly affect the folate content^75,85,106,292–303^.

#### Milling of cereals

Primary processing of cereals, particularly milling processes transforming cereals into more palatable and shelf-stable food ingredients, gives rise to significant folate losses because folates are not evenly distributed in grain fractions. The outer layers of the grain (the bran and the aleurone layer, the outermost layer of the endosperm, remaining attached to the bran during milling) and the germ are rich in folate, and they are generally separated during milling from the starchy endosperm, which is ground into flour^304–318^. Amounts of folate in refined wheat and rye flours decline by 21–89.5% and 27.7–83%, resp., in comparison to the whole grain ones^126,319–322^. Similarly, the folate levels in various barley and maize milled products, compared to whole cereals, decrease by 43.8–61.1% and 33–67%, resp.^319,320,323–326^. Commonly used oat flakes contain only 16% less folate compared to whole grains^327^. Folate losses are 46–79% and 27.3–55% in non-parboiled and parboiled white rice, resp., compared to brown rice. The folate decline in parboiled rice is generally lower, in contrast to the non-parboiled one, because a part of the vitamin diffuses from the vitamin-rich outer bran layer into the endosperm during the parboiling process, in which raw rice is soaked in water and partially steamed before drying and milling, and so it is retained during the following milling^313,319,325,328–333^. Considering the folate content, foods containing all components of the cereal grain (the ‘whole grain concept’) are more suitable for nutrition than those containing highly refined cereal products^304–306,334^.

#### Folate properties and stability; mechanisms of folate losses during food processing

Folates are water soluble and more stable in alkaline conditions with the lowest stability, unfortunately, in the pH range commonly encountered in plant foods (pH 4–6). Folates are sensitive to heat, atmospheric oxygen, UV radiation (e.g., present in sunlight), electron-beam radiation, and reducing sugars (such as fructose)^66,79,86,90,106,123,292,295,306,318,335–355^. Folic acid and 5-methyl-THF in aqueous solutions are photostable in the absence of oxygen^106,337,340,356–358^. Another B-vitamin, vitamin B_2_, riboflavin, as a photosensitizing compound^359^, gives rise to the oxidative cleavage of folic acid and 5-methyl-THF by visible light, which is absorbed by and yields excited states of riboflavin^356,360,361^. The degradation rate of folic acid in the presence of riboflavin depends on the pH, achieving the highest values at the pH around 6.2^362^. Iron and copper ions leaking from process equipment are prooxidative and hence catalyse the oxidation of folates^66,223^. Sulfite and nitrite, used as food preservatives, can cause degradation of folates, too^223,301,336^. Folates can resist heat degradation in anaerobic conditions, while they are degraded in the presence of oxygen; with folic acid, 10-formylfolate, and 5-formyl-THF being relatively stable vitamers and 5-methyl-THF and THF being very thermolabile ones. Folic acid is the most stable form. Hence, in general, the stability of formyl- and/or oxidized forms is much higher than that of methyl- and/or reduced ones, and the acidic environment accelerates the thermal decomposition^66,106,234,346,363–374^.

Two main mechanisms are involved in folate losses during food processing. The first one is leaching into the surrounding liquid, and the vitamin will be lost in any soaking or cooking water that is not consumed in the whole dish. The second one is oxidative degradation during heat treatment. The vitamin retention highly depends on the type of food, the method used, temperature, and process duration^102,106,130,134,162,180,295,296,363,365,375–390^.

#### Processing of vegetables and fruits

Boiling, steaming, and frying are estimated to cause average folate losses of 40–50%, 40%, and 15–30%, resp., in vegetables solely, and those of 25–30%, 30%, 30%, resp., in the total dish when cooking liquids are not thrown out^380,381^. Folate decline in vegetables during baking is estimated to be 20–35%^380–382^. For example, boiling, steaming, microwaving, and sous-vide resulted in folate losses of 36–62% and 25–56%, 9–57% and 10–30%, 51% and 17%, 41% and 23% in spinach and broccoli, resp.^102,162,379,384,386,391,392^. Non-leafy vegetables retain more folate during their boiling than do leafy ones^379,386,393^. Leeks, cauliflower, and green beans lost 26% and 28%, 8% and 10%, and 10% and 21% folate during steaming and blanching, resp.^394^. Similar changes concerning the relation between folate retention and processing methods (boiling, pressure cooking, steaming, and microwaving) were reported in frozen vegetables used for domestic cooking^393^. Freezing and thawing successively lead to tissue disruption and hence to a better release of folate. For instance, boiling fresh green beans and spinach caused no significant and 47% folate losses, resp., while that of frozen vegetables led to losses of 15% and 59%, resp., predominantly due to easier diffusion into the boiling water^379^. Likewise, blanching of fresh leeks, cauliflower, and green beans or frozen and thawed ones gave rise to folate losses of 28% or 85%, 10% or 65%, and 21% or 79%, resp., owing to leakage into the liquid during blanching^394^. Blanching of fresh vegetables before freezing reduced folate content by 12–35% in peas, 40% in cauliflower, 61% in cabbage, and 70% in spinach^395^. In another study, folate losses during vegetable blanching before freezing amounted to about 10% in broccoli, cauliflower, and green beans, 20% in peas, 26% in spinach, and only 1% in yellow beans^396^. Compared to fresh spinach, the folate amount declined by 38% in the frozen one, mostly due to the washing step and without any effect of the blanching step during the industrial freezing processing chain^397^. The total content of folate in vacuum-packed broccoli (crushed and mixed with water) decreased after heating at the higher temperature for a shorter time (90 °C, 4 min) less than after that at the lower temperature for a longer time (40 °C, 40 min), i.e., by 12% and 24%, resp.^234^. Folate levels in sweet corn cobs without bracts were reduced by 55%, 23%, and 20% by boiling, steaming, and microwaving, resp., compared to uncooked fresh corn^398^. Steaming in preference to boiling could be promoted as a means of saving the folate content of cooked green vegetables. Consumers choosing to boil vegetables should be strongly discouraged from doing so for prolonged periods if they would like to keep folate. In addition, minimalization of the cooking water and consumption it as soup or gravy will decrease the vitamin losses^384,399^. Likewise, other forms of cooking that minimize the direct contact with cooking water, such as steam blanching (instead of water blanching), steam pressure cooking, microwaving, and sous-vide are preferable to boiling in terms of folate retention^292,384,385,393^. Frying caused folate losses of 1–31% in drumstick, taro, bele, amaranth, and ota leaves, predominantly due to thermal destruction, while boiling caused those of 10–47%, mainly due to leaching, i.e., most lost folate was saved in the boiling water. Therefore, in terms of folate intake, boiling may be a healthier choice for cooking vegetables than frying, provided the cooking water is consumed together with the cooked vegetables^387^. Dried laver lost 8% of folate after toasting for 10 s^162^.

Sous-vide cooked, oven-baked, and boiled potatoes lost no, 37%, and 18–41% of their folate content, resp., compared to raw ones^384,385^. The presence or absence of potato skin had no significant impact on folate retention during boiling^384,385^. In other studies, folate content in boiled potatoes was reduced by 9% and 23% when they were unpeeled and by 23% and 39% when they were peeled^390,400^.

Retention of folates in green peas, broccoli, and potatoes cooked by different methods, stored, and reheated for use in modern large-scale service systems (e.g., hospitals) was also investigated. After-cooking storage at various temperatures (directly cooled or held warm and then cooled) and different periods followed by reheating caused no significant losses of folate^385^. On the other hand, folate content in three frozen vegetable-based ready meals declined by 7–37%, 11–45%, and 8–50% after reheating on a stove, in a microwave oven, and in a baking oven, resp. The study demonstrated that it is difficult to predict which reheating method is preferable regarding folate stability because no clear pattern in folate retention between different heating methods was seen^401^.

Folate losses could be expected during the production of fruit and vegetable juices. It includes various technological steps, among others, separation of pomace and pasteurization or, in the case of juice concentrates, also thermo-vacuum evaporation. The production process of sea buckthorn juice and juice concentrate resulted in folate losses of 19% and 25%, resp., compared to berries^402^. Berry juices (golden raspberry, red raspberry, blackberry, blueberry, cherry, and strawberry) contained 7–22% less folate than berries^37^. Folate contents in fresh, non-pasteurized juices were reduced by 11–40% in leafy vegetables (beet greens, turnip greens, Romaine lettuce, and carrot greens), by 32–49% in root vegetables (beet, turnip, and carrot), and by 49% in broccoli compared to the initial vegetables^403^.

Rosehips, rich in folate and ascorbic acid, have traditionally been used as a health food supplement in many European countries. They are not often consumed fresh, and therefore, air drying to produce a stable product is a crucial step. The degradation of folate was shown to be affected by temperature and dependent on the drying time – shorter drying time at a higher temperature can limit vitamin decomposition mediated by thermal degradation. The cutting of rosehips into slices reduced the required drying time from 11 h to 100 min and decreased average folate losses from 27% to 18% for whole rosehips and slices, resp., compared to fresh rosehips. When sliced rosehips were dried, an increase in temperature from 70 °C to 90 °C shortened the necessary drying time from 160 min to 105 min and lowered folate losses from 21% to 13%. The levels of ascorbic acid seemed to follow the same pattern as the folate levels during drying; a high content of ascorbic acid could provide possible protection of folate from degradation^404^. Folate content was determined in sultanas after rack, ground, trellis, or natural drying of vine fruits. The folate levels differed, depending on the drying method, the highest being in emulsion-rack dried sultanas^70^.

#### Processing of legumes

Legumes are usually processed before consumption, and their processing may cause losses of folate^405–408^. Folate content in boiled (heated to boiling temperature and then simmered for 2 h) soaked peas and chickpeas decreased by 55% and 47%, and that in pressure-cooked (for 20 min) ones by 49% and 38%, resp., compared to raw legumes. Leaching was the main reason for the vitamin loss because nearly all the lost folate was found in the water used for soaking and heat processing. Higher folate retention in pressure-cooked legumes can be attributed to the shorter exposure to heat^409^. On the other hand, in navy beans, pressure cooking caused higher folate reduction than ordinary cooking. Folate stability was higher in the beans cooked in a water-oil mixture than in water. Folate declines were lower in non-soaked than in soaked navy beans during the following cooking^410^. Fresh kidney beans boiled for 10 min and dried red beans boiled for 30 min without soaking lost 14% and 19% of folate, resp.^162^. Effects of boiling on folate retention were evaluated in soaked mung beans, adzuki beans, cowpeas, faba beans, peas, and common beans. Folate losses in the boiled pulses due to heating degradation and leaching depended on the pulse variety and ranged from 18% to 36%, with an average of 24%, compared to unprocessed ones^411^. Boiling of soaked lentils and soybeans for 25 min resulted in a folate decline of 57% and 5%, resp.^102^. Blanching before canning decreased folate amounts by 10% and 21% in soaked faba beans and chickpeas, resp. The folate content in the germinated faba beans declined by 32% after boiling, mainly due to the leaching and not degradation, as approximately 90% of the lost folate occurred in the cooking medium (which is also consumed as nabet soup). After deep-frying falafel balls made from the soaked faba bean paste, folate losses of 10% due to the heat treatment were observed^412^. In West Africa, cowpea seeds are usually prepared by using two different methods. The first consists of directly boiling the seeds in water for 1 h, and the second involves a pre-soaking step for one night followed by boiling in water for 25 min. Both methods resulted in a similar decrease in total folate concentration, the former by 38% and the latter by 43%. However, the latter is recommended because of improved folate bioavailability. During a pre-soaking step, enzymatic interconversion of folate vitamers takes place in favour of 5-methyl-THF, which is considered the most bioavailable of all the vitamers present in seeds^413^. Folate losses of 26% and 29%, compared to raw soybeans, occurred during the preparation of tempeh, involving soaking, dehulling, boiling, and fermentation, and that of soymilk, involving soaking, blanching, milling, and homogenization, resp. Deep-frying of tempeh and ultra-heat treatment of soymilk caused a folate decline of 21% and 14%, resp., in comparison to unprocessed products^414^. Likewise, the preparation of tofu, involving soaking, milling, boiling, coagulation, and pressing, led to 60% losses of folate compared to raw soybeans. Most of the lost folate was found in the whey after pressing^415^. Similar folate reduction during the processing of raw soybeans into tempeh and tofu was also observed in another study^416^. In one study, tempeh contained 68% more folate than the starting raw soybeans, apparently owing to using a different fungus strain in the fermentation step with much higher folate synthesis capability than in other cases^417^. Due to naturally high folate amounts, soybeans, tempeh, tofu, and soymilk are good dietary sources of folate, despite the losses during preparation^414–416^.

#### Breadmaking and other processing of cereals and cereal products

Breadmaking, a common process to prepare cereals for consumption, involves many variable factors, which can affect the folate levels in the end-product^418^. It is presumed that folates in bread derive not only from flour but also from yeast. A bread made using yeast usually contains more folate than the flour from which it was made, even though some folate losses occur during baking^126,321,372,419–421^. Yeast has a high content of folate, but also the ability to synthesize the vitamin during fermentation, and this may compensate for folate losses during baking. The sourdough fermentation is a traditional practice, especially in rye bread making, to improve the sensory quality and shelf-life of bread. A sourdough starter consists of lactic acid bacteria, whose contribution to enhanced folate levels, in contrast to yeast, is negligible^372,418,422–424^. Folate amounts in white bread differed up to 3.2-fold depending on a combination of various factors, such as the wheat flour extraction rate, leavening agents (baker’s yeast or baker’s yeast with sourdough), and prebaking and baking conditions (different sets of time and temperature)^425^. The white wheat bread had an 11% lower folate amount than the whole-grain one^195^. The folate content was 80% and 40% higher in the dough after fermentation and in the wholemeal rye bread, resp., compared to the starting flour, and similarly, 109% and 38%, resp., in the wheat bread. About 22–25% and 25–34% of the folate in the fermented dough was lost during the baking of rye and wheat bread, resp.^316,372,419,426^. Rye bread baked using lactic acid bacteria fermentation contained 31% less folate than those using yeast alone or yeast with lactic acid bacteria for fermentation^372^. The use of baker’s yeast during the baking procedure considerably increased (2.1–2.5-fold) the folate content in the wheat bread in comparison to the use of baking powder as a leavening agent^195,372^. Steamed whole-grain wheat bread contained 16% more folate than the oven-baked one^195^. During breadmaking, there was a decrease of added folic acid from fortified flour to bread stage by about 20% and 22% for wheat and rye bread, resp.^420,427^. Folic acid losses in fortified wheat breakfast rolls, Baladi bread, white pan bread, wholemeal pan bread, white baguette, and brown soda bread due to baking amounted to 19–25%, 15%, 24%, 32%, 22%, and 26%, resp. Consequently, folic acid averages of around 10–25% in the flour are necessary to compensate for the losses during baking and to achieve the required folic acid values in fortified bakery products^302,426,428,429^. The effect of breadmaking on the retention of two fortificants, folic acid and 5-methyl-THF, in wholemeal rye bread was compared. Breadmaking resulted in losses of 24% and 65% for folic acid and 5-methyl-THF resp. Retention of 5-methyl-THF fortificant during breadmaking varied depending on the bread size (so on baking time) and it was only 50% of that of folic acid in breads of the same size^127^. The amounts of folic acid and 5-methyl-THF used as fortificants in wheat bread were reduced by 10% and 47%, respectively due to breadmaking^430^. A loss of 5-methyl-THF fortificant in wheat bread baked in a commercial bakery amounted to 71%^431^. The influence of breadmaking on the folate content in white and whole-grain bread fortified with 20 g/100 g and 40 g/100 g fresh vegetables, either Swiss chard or spinach, as a natural source of folate, were studied. Although the magnitude losses of folate content of raw materials (wheat flour, wheat bran, dough, baker’s yeast, and vegetables) due to the heat treatment during breadmaking were about 45%, the fortification increased the total folate content by up to 190% and 100% in white and whole-grain bread, resp., without adverse effects on sensory properties, such as odour and taste, or overall consumer acceptance of the vegetable-fortified bread^432^. Injera is an Ethiopian fermented flatbread usually made from the whole grain gluten-free cereal tef. Both main processing steps during traditional injera preparation, i.e., fermentation mostly by lactic acid bacteria and baking, led to folate reduction. The folate content in injera was, on average, 32% lower compared to tef^433^.

Tarhana, a traditional Turkish dried soup based on a fermented mixture of wheat flour and yoghurt, is prepared through lactic acid fermentation, initiated by yoghurt or sour milk. The fermentation for 2–4 days resulted in a folate increase of 21–26%. Drying of tarhana brought about folate losses of 6%, 10%, and 17% at temperatures of 50 °C, 60 °C, and 70 °C, resp.^434^.

Nixtamalization is a process for the preparation of maize, which is used for the production of tortillas. It involves cooking and steeping dried corn in hot water with calcium hydroxide, discarding steeping liquids, and washing with subsequent removal of the pericarp (hulls). The resulting product is called nixtamal. Fresh nixtamal is wet-milled to make masa (nixtamal dough), which is formed into tortillas and baked (the traditional method), or it can also be dried and ground to make corn masa flour (nixtamalized corn flour), which is mixed with water to prepare masa that is used for baking tortillas. Nixtamal and corn masa flour can be fortified with vitamins and minerals^435,436^. Fortified tortillas made from masa produced through the traditional nixtamalization and wet-milling process, where folic acid was added to nixtamal before milling, contained 15%, 33%, and, in one study, even 80% less folic acid compared to the folic acid amount added to nixtamal (theoretical fortified level). No significant differences in folate levels were found in prebaked masa and baked tortillas. Baking as a high temperature/short time process (usually 290–300 °C for 42–50 s) had a minimal effect on folate content. It was observed that the commercial production step resulting in the greatest folate loss was the holding of hot, freshly ground, fortified masa (for 0.5–4 h) before baking. The losses in commercial masa increased significantly with prebake masa holding time. It was supposed that folic acid losses could be owing to utilization by lactic acid bacteria, which are naturally present in masa and whose count increased in masa during storage^435–437^. This assumption was not confirmed in an experiment with bacteria isolated from the dough (corn masa) samples from six commercial tortilla mills. Sterile fortified masa inoculated with bacteria, held at 56 °C for 3 and 6 h, replicating the conditions of freshly milled masa as held before baking in commercial tortillerias, showed folic acid losses of 66–79% in the first 3 h of incubation. Losses to the same extent were found in control non-inoculated sterile masa incubated under identical conditions after 3 and 6 h. In addition, the losses were comparable to those reported in the above-mentioned studies for the time between masa fortification and tortilla baking^438^. The decline in folic acid was not owing to bacteria. The traditional method produces substantial heat during the grinding of nixtamal to masa and involves the holding of hot masa until it is used. The combination of the high moisture content of masa and high masa holding temperatures before baking is the likely cause of folic acid chemical degradation when using the traditional method. Encapsulation of folic acid may help mitigate the problems^435,438,439^. On the other hand, fortified tortillas and tortilla chips prepared from masa made by mixing water with fortified corn masa flour lost 13% and no folic acid during tortilla baking and chip frying, resp., compared to fortified masa flour^439^.

During roasting barley malt for 20 min, the folate content was not affected at a temperature of 100 °C but declined continuously with increasing temperature up to 200 °C, at which folate was completely degraded. Barley malt may be used in several food products, and therefore, it would be beneficial to apply its pale form, as the coloured types are treated at higher temperatures resulting in lower folate content^440^. Extrusion decreased folate amounts by 26% and 28% in non-germinated and germinated rye grains, resp., compared to unprocessed grains^441^. Extrusion processing of corn and wheat flour blend and rice flour alone fortified with folic acid led to a folic acid decrease of 10% and 63%, resp., in extruded rice-shaped kernels^442^.

The average folate losses caused by cooking brown and white rice reached about 40% and 48%, respectively^330,443^. Rinsing before cooking had almost no effect on folate levels in brown rice but removed 73% and 88% of folate in fortified parboiled and non-parboiled white rice. Rinsing did not reduce detrimental inorganic arsenic in whole grain (brown) rice and eliminated 5–13% and 13–19% of arsenic in parboiled and non-parboiled white rice, resp. Cooking in variable amounts of water decreased folate contents, with increasing water excess, by up to 65–70% at a water-to-rice ratio of 10:1 for all three rice types (less in brown rice), but at the same time, efficiently reduced the quantity of inorganic arsenic by up to 60%, 70–83%, and 45–54% in cooked rinsed brown, parboiled, and non-parboiled white rice, resp.^444^. Losses of folic acid in fortified rice cooked by different methods (e.g., stir-frying and boiling, microwaving, and boiling) amounted to 8–66%, on average. The retention of folic acid seems to be more affected by the type of fortification (e.g., coating, cold extrusion, hot extrusion, parboiling, and sonication) than by the cooking method^445–452^.

Folate declines due to boiling were 36%, 15–30%, and 4–6% in spaghetti, white and yellow Asian noodles, and instant Asian noodles, resp.^162,453^. Preparation of fortified white and yellow Asian noodles, including dough kneading, cutting, and drying, led to minimal (1.3%) folic acid losses compared to fortified wheat flour. Preparation of fortified instant Asian noodles, involving additional processing steps (steaming, frying, and draining), led to a loss of 32%. Compared to the starting fortified flour, total folic acid losses after boiling all three styles of fortified Asian noodles amounted to about 41% in white and yellow and around 43% in instant noodles^454^. In other studies, no changes in the content of folic acid, which was used for the fortification of wheat flour, during the main four stages of instant fried Asian noodle manufacturing (mixing, sheeting and cutting, steaming, and frying) were observed^455,456^. A comparison of retention of folic acid and 5-methyl-THF used for fortification of flour during the noodle-making and the following boiling showed a very low, no significant losses in folic acid during both processes whereas a loss of 28% was found during the noodle-making, compared to fortified flour, and that of 57% during the noodle boiling, compared to fresh noodles, was observed when 5-methyl-THF was used as the fortificant. Compared to the fortified flour, the boiled noodles contained 69% less 5-methyl-THF fortificant^457^. Commercial unfortified durum wheat pasta lost 51% folate after boiling. In commercial durum wheat pasta fortified with folic acid, folic acid content declined by 72% after boiling^458^. The effects of two preparation methods on folate losses in rice noodles fortified with folic acid were examined – raw noodles (i.e., extruded kneaded dough) were boiled or steamed. The folate losses observed after the boiling (process A) or steaming (process B) of raw noodles, drying of fresh noodles prepared by either of the two types of noodle processing methods, and cooking (boiling) of dried noodles prepared using processes A or B were 42% or 20%, 0%, and 72% or 53%, resp., compared to the initial content of added folic acid in fortified rice^459^. In another study, the influence of rice flour particle size (≤63, 80, 100, 125, and 140 μm) on the retention of folic acid fortificant during rice noodle processing was analysed. Compared to 100% of folic acid in fortified rice flour, the amount of folic acid in the five types of rice noodles decreased by 50–56% after boiling the raw noodles and by 7–13% after cooking (boiling) the dried noodles before consumption. The reduction in the particle size of rice flour led to a decline in the losses of the fortificant^460^.

#### Processing of eggs, milk, and meat

Eggs lose 0%, 19%, 2–39%, 11–47%, and 10–50% folate, resp., due to poaching, scrambling, boiling, frying, and baking^92,100,102,104,162,329,375,376,382,383,443^. In milk, heat-induced folate decrease amounted to 8–10%, 4–20%, and 42–45% during pasteurization, ultra-heat treatment, and sterilization, resp. Modern technologies reducing oxygen levels in the milk before ultra-heat treatment increase folate retention in the processed milk^75,149,228,461–463^. Folate content declined by 27%, 35%, 41%, and 52–63% in the beef after boiling, in the pork loin after pan-broiling, in the chicken breast after boiling, and in the mackerel after shallow-frying, resp.^162^. Steamed mackerel (i.e., the common form sold) lost 24% of folate during frying in soybean oil; the estimated total loss of folate in the mackerel by steaming and frying was 74%^443^. The influence of stewing and roasting on folate content in white and dark, fresh or frozen, chicken meat was also studied^464^. Sous-vide (60 °C/75 min) and steaming (100 °C/30 min) did not significantly affect folate amounts in chicken liver, whereas another sous-vide (75 °C/45 min), grilling without oil addition (200–220 °C/4 min), grilling with oil addition (170–200 °C/6 min), and baking (180 °C/30 min) decreased them by 16%, 9%, 22%, and 42%, resp., compared to raw liver^465^. Manufacturing of fortified sausages, including cooking in a steam oven at 72 °C, did not influence the content of added folic acid^466^.

#### Food preservation techniques – canning, ionizing irradiation, and high pressure processing

The effects of industrial canning on folate content in green beans were investigated. Compared to fresh vegetables, folate content lessened by 10% in green bean cans (30% in beans alone, but most of the lost folate was retained in the covering liquid), mainly owing to the sterilization step with no significant impact of washing and blanching steps during the canning chain^397^. Canning reduced folate by up to 40% in table beets with increasing processing time and temperature, while it did not cause any significant folate amount changes in green beans, compared to unprocessed vegetables^467^. Industrial canning, including soaking, blanching, and autoclaving, resulted in losses of 0–20% and 24% in faba beans and chickpeas, resp., in comparison with raw legumes. Soaking of legumes brought about folate increase (probably due to enzymatic de novo synthesis from initiated germination), blanching, and mainly autoclaving led to folate decline. The folate lost from legumes during autoclaving was recovered in the canning medium^412,468^. In cans, folate concentrations are usually equilibrated between the vegetables and the covering liquid^379,397,469^. The folate content in strawberry jams was 9–16% less than in the initial frozen fruit^49^.

Ionizing radiation (accelerated electrons, gamma rays, and X-rays) is used as a non-thermal preservation technology for extending shelf life and increasing the safety of food^348,470^. Electron-beam irradiation (2 kGy) decreased folic acid levels by about 20–30% in hamburgers and sausages fortified with folic acid^471,472^. Wheat flour fortified with folic acid showed no significant loss in its folic acid content following electron beam irradiation at doses of up to 1 kGy (doses required for disinfestation). Around 30% of folic acid was degraded when fortified flour was irradiated at doses of 5 and 10 kGy. The higher stability of folic acid in flour than in meat products is explained by differences in moisture. Non-solubilized folic acid in dry materials is not sensitive to irradiation treatment, while it is easily degraded in aqueous solutions^348,349^. Gamma-irradiation at doses of 1, 2, and 5 kGy did not influence the folate amount in watercress, whereas at a dose of 2 kGy, folate content declined by 34% in buckler sorrel. Different sensitivities were likely because of the plant matrix effect^179^. Folate amounts in gamma-irradiated baby-leaf spinach declined with increasing dose of irradiation from 0.5 to 2 kGy reaching losses of about 24% at the highest dose, irrespective of whether the treatment took place in the air or nitrogen atmosphere^470^.

High (hydrostatic) pressure processing is a novel technique for the preservation of food products in a gentle way, allowing better retention of food sensory and nutritional quality; it inactivates microorganisms in foods due to permeabilization of cell membranes^394,473–477^. Effects of high pressure processing on folate stability were investigated in model solutions as well as in vegetables (carrot, asparagus, green beans, yardlong beans, winged beans, leeks, cauliflower, and broccoli) and fruits (orange, kiwi, and papaya). Depending on processing conditions (pressure-temperature-time combinations), various, sometimes marked, folate losses were observed^365,369,374,394,473,475,478–481^. Folates during that processing were shown to be more stable, e.g., in fresh-cut papaya, freshly squeezed orange juice, and kiwi puree; all those fruits are naturally rich in ascorbic acid, which may protect folates against pressure and heat degradation^365,477,479^. Folate stability during high pressure processing is comparable to that during heat treatments. Though high pressure processing is generally considered to lead to better preservation of vitamins, compared with thermal treatment, this obviously is not the case for folates^106^.

#### Storage

Folate losses can occur during the storage of foods, depending on the storage conditions and duration. Green beans, leeks, and cauliflower lost no, 15%, and 25% of folate, resp., during storage in a refrigerator at 4 °C for 24 h^394^. No folate losses occurred in untreated green beans, yardlong beans, and winged beans during storage in a refrigerator at 4 °C for 10 days, while after high-pressure treatment preceding the storage, profound folate degradation happened, which was positively proportional to the increase in pressure and extending of holding time during treatment^473^. Fresh spinach commercially packaged in polyethylene plastic bags was stored at 4 °C, 10 °C, and 20 °C °C for 8, 6, and 4 days, resp. Based on the visual colour and appearance, spinach was commercially unacceptable after those storage times (shelf-life values). Folate levels decreased with increasing storage time at approximately the same rate for each temperature, reaching a loss of about 47% at each temperature and shelf life compared to the initial folate amount. Therefore, producers and retailers should maintain storage temperatures as low as possible to minimize the vitamin losses in fresh spinach. Consumers should keep fresh spinach refrigerated and use it as close as possible to the time at which it was purchased^482^. Folate content in frillice, rocket, and iceberg lettuce was reduced by 2–40% after storage at room temperature (22 °C) in regular light after 2–4 h to simulate the conditions in lunchtime restaurants, depending on whether samples were stored as whole leaves, or small torn or cut pieces. Storage of lettuce in a refrigerator at 4 °C for 8 days led to folate losses of 14%^69^. No significant changes in folate content occurred in choy sum during storage at 4 °C in the dark for 3 weeks^182,483^. Storage of watercress and bucker sorrel in polyethylene bags at 4 °C for 7 and 12 days, resp., gave rise to a loss of 37% in the former and no alteration in the latter in folate content^179^. Storage of fresh sweet corn cobs in bracts at room temperature (25 °C) or in a refrigerator (+4 °C) caused folate reduction of 32% and 24% in 3 days, and that of 54% and 55% in 7 days, resp.^398^. The percentage of folate losses in strawberries during refrigerated storage at 4 °C amounted to 21%, 42%, 55%, 78%, 88%, and 93% on days 1, 2, 3, 4, 5, and 6, resp., compared to fresh fruits (day 0). Therefore, strawberries should be consumed within a day or two after harvest before the folate losses reach more than 50%^178^. In another study, the folate content in fresh strawberries declined by 16% and 29% during 3 and 9 days of storage, resp., at 4 °C in the dark; after 9 days, strawberries were considered not fit to be eaten. On the other hand, the storage at room temperature (20–25 °C) in daylight, mimicking the procedure of commercial retailing, led to folate losses of 27% and 38% after 1 and 3 days, respectively^49^. Strawberry puree lost no, 13%, 43%, and 84% of initial folate content after 1, 2, 3, and 4 days, resp., storage at 7 °C in the dark^484^. Potatoes are often stored at low temperatures for several months before processing. Folate concentrations increased in tubers stored in the cold. The extent of the increase, which seems to be genotype dependent, was about 2-fold at 9 °C after 4 months or up to 1.8-fold at 4 °C after 7 months^151,152^.

Storage of blanched vegetables at −20 °C for 12 months did not affect folate content in peas, cauliflower, cabbage, and spinach and that for 6 months in green faba beans^395,468^. In another study, the 5-methyl-THF content in blanched vegetables decreased with the time of frozen storage at −18 °C by 98% in cauliflower, 24% in broccoli, 39% in peas and spinach just after 3 months, and by 82–98% in all of them after 6 months. In green and yellow beans, significant losses of 75% and 95%, resp., were observed no earlier than after 9 months of frozen storage^396^. No loss of 5-methyl-THF was detected in spinach, broccoli, potatoes, strawberries, apples, oranges, and bananas frozen at −60 °C after storage for 12 months^485^. The fresh kernels of sweet corn stripped from the cobs stored at −20 °C lost 62% of folate after 4 months^398^. Frozen products can lose folate during storage due to oxidation, in contrast to canned products, which can lose more during the initial thermal treatment, but then are relatively stable because of the lack of oxygen^293^.

Folate losses reached values of 76.4–79.7% in glass-bottled tomato juice after storage in the dark for twelve months, irrespective of storage temperature (8, 22, and 37 °C)^486^. Folic acid degradation in fortified vitamin juices during long-term storage was studied. The juices were stored in the dark and light (500 lux for 10 h/day) in light-transmissive (clear PET and glass) and non-transmissive (brown PET and cardboard) packaging at 18 °C, reflecting common storage conditions, e.g., at a supermarket. Average decreases in folic acid concentrations of 36% (dark) and 39% (light) after 6 months and 47% (dark) and 50% (light) after 12 months of storage were observed^487^. Natural folates and added folic acid in fortified orange juice stored below 8 °C in the dark were stable during shelf life for 35 days (best before date) and during one-week simulated household consumption. The high endogenous ascorbic acid content in the juice might have prevented oxidative degradation of natural folates and added folic acid. This suggests that orange juice may be considered a good source of natural folate regarding content and stability during storage and a suitable vehicle for folic acid fortification^488^. Sea buckthorn juice was stored in the dark under two household storage conditions (6 °C and 25 °C) and accelerated aging conditions (40 °C) for up to 7 days. The folate content was almost unchanged during the storage at 6 °C after 7 days. The juice showed folate losses of 5% at 25 °C and 17% at 40 °C after 7 days of storage^402^.

When wheat grains and whole-grain powder were stored in closed paper bags at room temperature for 8 months, the folate loss occurred earlier in powder (after 2 months of storage) than in the grains (after 4 months of storage). The average folate losses in grains and powder after 6 months of storage were 17% and 28%, resp., indicating that folates were more stable in the grains than in powder up to 6 months of storage. The 8-month storage led to a more extensive folate reduction both in the wheat grains (26%) and the whole-grain powder (30%)^321^. Storage of cereal and pseudocereal wholemeal flours in paper bags at 20 °C and 50% relative humidity for 3 months caused a folate decrease of 45%, 37%, 19–38%, 41%, and 23% in wheat, rye, amaranth, buckwheat, and quinoa, resp.^130^. Factors influencing folic acid content in fortified wheat flour were studied too: packaging (paper bags or multilayer aluminium/PET bags), temperature (25 °C or 40 °C), relative humidity (65% or 85%), and duration (6 months). In flour packed in multilayer bags (non-permeable to oxygen and humidity), no significant folic acid losses were observed after 6 months, irrespective of temperature and relative humidity. In flour packed in permeable paper bags, folic acid content decreased by 17–19% after 3 months when flour was stored at 65% relative humidity, regardless of storage temperature. At 85% relative humidity, folic acid decreases of 21–22% at 25 °C and 40–49% at 40 °C were found after 3 months of storage. In flour packed in paper bags and stored for 6 months, folic acid losses of 15–20% at 25 °C and 20–22% at 40 °C during storage at 65% relative humidity and those of 22–27% at 25 °C and 47–53% at 40 °C during storage at 85% relative humidity were observed. The observed folic acid losses in fortified flour packed in paper bags were most likely due to oxidative degradation. Therefore, the choice of suitable flour packaging, which is not permeable to both oxygen and moisture, is of critical importance in limiting losses of added folic acid, and it must be taken into account when planning a fortification program in countries with a tropical environment. Co-fortification with or without ferrous sulfate did not have any significant effect on the folate retention in wheat flour fortified with folic acid, irrespective of storage conditions and packaging^489^. There was no significant decrease in folic acid fortificant content during the six-month shelf life of fortified corn masa flour^439^. The average folate losses in rice (brown and milled) due to storage in paper bags for 1 year reached nearly 23%^330^. Storage of fortified rice under accelerated conditions (fluorescent light at 40 °C) in different packaging for 3 months caused no significant changes in folic acid content^446^. Folic acid losses of 0–18% and 24–43% after 3 and 9 months of storage under typical tropical conditions (40 °C and 60% relative humidity), resp., were observed in rice extruded products prepared from rice flour fortified with folic acid and various iron compounds. Increased iron concentration levels resulted in faster degradation and more loss of folic acid^490^.

Storage of Baladi bread in polyethylene bags at ambient room temperature (about 20 °C) in the dark (cupboard) according to household practice or chilled (about 5 °C) for 48 h (i.e., shelf-life) did not significantly affect folate content, compared to bread after baking^421^. Storage of different rye breads at −18 °C for 2 weeks did not influence folate contents. However, during prolonged storage, folate contents gradually dropped, reaching 25% and 38% losses in the bread leavened with baker’s yeast and in the bread fermented with sourdough, resp., after 16 weeks, likely due to air oxidation. Higher folate content reduction in the bread made using sourdough was explained by its acidic pH, which is less favourable for folate stability, as mentioned above^424^. Losses of fortificants in fortified wheat bread stored in paper bags at room temperature (21 °C) for 7 days amounted to 3% for folic acid and 82% for 5-methyl-THF^430^. Folic acid was stable in fortified wheat breakfast rolls for 90-day storage at −20 °C^429^. Storage of fortified tortillas and tortilla chips in sealed low-density polyethylene bags at 22 °C and 65% relative humidity for 2 months, common shelf life for these products, led to a folic acid decrease of 13% and 9% in the respective products^439^.

The vacuum-packaged tortillas and the vacuum-packed freeze-dried broccoli au gratin were stored either on Earth or aboard the International Space Station at room temperature for 880 days. The folate contents declined and were not significantly different in flight and ground samples during the storage. Folic acid levels in tortillas were about 15% and 45% lower after 13 and 880 days, resp., compared to the initial analysis. A folate decrease in broccoli amounted to about 15% and 22% after 13 and 880 days of storage^491^.

Folate was stable in cold stored eggs (4 °C) for four weeks^492^. Similarly, no changes in the folate content were observed in eggs stored at refrigerator temperature (4–7 °C) or room temperature (18–20 °C) for 27 days (i.e., from the date of laying to the best before date). The same was confirmed for novel eggs enriched with natural folate through the addition of supplemental folic acid to the hen’s feed^493^. The folic acid level in sausages fortified with folic acid was retained after 3 months of refrigerated storage (4 °C)^466^. No alteration or a decline of 81% occurred in folate amounts in whole-milk powder during storage in the nitrogen or oxygen atmosphere, resp., for 57 days. Similarly, in skimmed milk powder stored at 37 °C, folate content decreased by 13% and 30% in nitrogen and by 86% and 88% in oxygen atmosphere after 25 and 105 days, resp. Exclusion of oxygen from the package is necessary to prevent folate degradation during the long-term storage of milk powders^494^. Folate losses in ultra-heat treated milk packed in Tetra Pak stored at 24 °C amounted to 11% and 32% after 12 and 20 weeks, respectively^463^.

#### Enhancement of folate content through processing

There are food process techniques that can elevate the content of folate. Before cooking pulses, soaking is a common processing step employed to soften and make the seeds more digestible. Soaking, probably due to enzymatic de novo synthesis activated upon the initiation of germination, increased folate content by 46%, 28%, 16%, 65%, 81%, and 13% in mung beans, adzuki beans, cowpeas, faba beans, peas, and common beans, compared to raw pulses. In addition, some folate diffused into the soaking water; it represented, on average, 15% of the total folate enhancement during soaking^411^. In another study, an increase in folate content during soaking in faba beans and chickpeas by 39–51% and 51–66%, resp., was observed^412^. Folate levels increased in soybeans by 10–15% after soaking for 12 h and then declined likely owing to dissolution in water^495^. The behaviour of folate during soaking depends on various factors, e.g., duration, seed-to-water ratio, temperature, and to a great extent on the legume species, which differ in their germination capacity^413^.

Germination could be more beneficial than soaking to enhance the production of folates in seeds for human consumption^407,496^. Germination of plant seeds is a biological process used to obtain a typical flavour and texture in foods and a natural way to increase folate levels. It has been applied for a long time^302,497,498^. Germination of faba beans, chickpeas, brown lentils, white beans, black-eyed peas, soybean, mungbean, and cowpea resulted in an up to 1.77, 2.4, 3.1, 2.8, 2.6, 3.7, 4.3, and 2-fold increase in folate content^406,412,496,499–502^. Therefore, germination of legumes can be recommended to produce foods with enhanced folate content. For example, household preparation increased the folate levels in germinated faba bean soup (nabet soup) by 100% and in bean stew (foul) by 20%, compared to raw beans^412^. The novel industrial canning process for dried faba beans, which newly involved pre-germination of soaked dried seeds, led to a 52% higher folate content in the novel product compared to the conventional canned beans^468^. In germinated rye, wheat, and barley, the folate increased by up to 5.3, 5.7, and 7-fold, resp.^316,421,423,440,441,497,503,504^. Germinated cereal grains could serve as functional ingredients for the breadmaking industry. It was shown that oven-drying of germinated wheat grains at 50 °C did not affect the folate content, so it did not decrease the improved nutritional value of germinated grains^421,503^. By the addition of germinated wheat flour to the native one, bread with 66% more folate compared to conventional Egyptian baladi bread could be prepared^421^. Germination enhanced folate content in pseudocereals, namely by 21% and 26% in amaranth and buckwheat, resp.^134^. Increased folate levels were also observed during the germination of maize seeds^505,506^.

Beers contain various amounts of folate owing to the differences in the brewing process and the choice of raw materials, which influence not only the sensorial profile but also the level of health-positive compounds, including folate. In small- and large-scale brewing, the folate content increased during mashing, decreased after wort boiling, and increased during fermentation. Large-scale brewing showed a decline in folate between the end of maturation and the final bottled beer because of operations that do not occur in small-scale brewing, such as filtration, pasteurization, and dilution to the desired gravity with deoxygenated water^302,440,507–515^.

In wines, folate amounts vary, like in beers. There was no significant difference between red and white wines in the folate content range. The chemical composition of wine is determined by two factors: the initial grape must and the fermentation by yeast. The folate content of wine is generated primarily by the yeast during fermentation rather than being present at appreciable levels in the starting grape must. There is a large variability in the ability of the different yeast strains to produce folate^516^.

Owing to fermentation, folate content rises not only during breadmaking, as reported in this paper, but also during the production of fermented dairy products. For example, yoghurt usually contains 2-fold higher amounts of folate compared to the original milk, dependent on starter cultures used (bacteria species and strains)^302,517–520^.

Folate content in plants may be increased by stimulation of folate biosynthesis. Enhanced folate accumulation stimulated by red light irradiation and amino acid addition in wheat seedlings, phenylalanine addition in hydroponically cultivated spinach, cool and warm white light in Lamb’s lettuce leaves, and salicylic acid in coriander foliage and foxtail millet panicles were reported^504,521–526^.

Changes in folate content during ripening (i.e., different maturity stages) were studied in corn^398,527,528^, cowpea leaves^399^, winged beans^529^, potato tubers^530^, faba beans^468^, tomato^4–6,181,486,531–534^, avocado^531^, strawberries^49^, banana^531^, Australian green plum^535^, and papaya^38,531^. Treatment by exogenous ethylene, as a common postharvest practice to trigger the ripening of mature green fruits before placing them on the shelf, caused a 24% and 51% folate increase in tomatoes and bananas, resp., a 26% folate decrease in papayas, and no change in avocados, compared to non-treated fruits^531^.

The content of folate in eggs was affected by the rearing system; eggs from the organic farming system contained significantly more folate (by about 36%) than those from the free range, barn, and cage systems, in which the folate contents were comparable^92^. In another study, significantly higher folate levels were found in eggs from the free range system than from the barn one (by 58%)^493^. There was no significant difference in amounts of folate in eggs from three different breeds of hens raised on farms fed with three different feeds (one organic and two conventional)^104^. Supplementation of laying hens by feeding with folic acid brought about a 2–3-fold increase in egg folate content. Moreover, folic acid from feed was converted to natural folate vitamers, especially 5-methyl-THF^492,536–542^. Folic acid in total egg folate content represented at most 10%, a level which would be converted into biologically active folates by humans after ingestion. Folate-enriched eggs produced in this way could offer an alternative without the safety concerns related to folic acid-fortified foods^493,536,542,543^.

#### Food ingredients influencing folate stability

Some food ingredients and natural compounds may influence the stability of folates. Ascorbic acid (vitamin C) protected folates, naturally present in foods or folic acid and 5-methyl-THF added as fortificants, against degradation by heat, oxidation, and ultraviolet radiation during processing and storage in model systems and food products. The addition of ascorbic acid could be considered as a strategy for preventing folate degradation during processing^193,346,365,371,430,431,457,475,477,544–549^. Vitamin C and, to a higher extent, vitamin E added to egg yolk preserved 5-methyl-THF from thermal oxidative degradation during yolk thermal pasteurization or spray-drying^347^. The thermal stability of 5-methyl-THF increased in skim milk due to the presence of casein and folate binding protein, and in soymilk due to the presence of phenolic antioxidant compounds^545^. Tannic acid, a polyphenolic compound used as a food additive, improved the photostability of folic acid against ultraviolet light in solution and in gummy, a common delivery system for vitamins in supplements^550^. Similarly, the photodecomposition of folic acid by ultraviolet radiation was inhibited or delayed in varying degrees by natural phenolic compounds, such as hydroxycinnamic acids (e.g., caffeic acid, ferulic acid, and *p*-coumaric acid), flavonoids (e.g., quercetin and epigallocatechin gallate), stilbenes (e.g., resveratrol), etc., with caffeic acid being the most effective. The findings are useful for the protection of food and beverages against undesired effects of light exposure, i.e., for preventing premature quality loss and for the co-encapsulation of folic acid with those antioxidants as an effective way to protect the vitamin B_9_^551–553^. Also using green tea-enriched extracts containing epigallocatechin gallate and epigallocatechin would be a simple and relatively inexpensive method to preserve 5-methyl-THF against air oxidation^554^.

Folic acid loss occurs in solutions upon heating in the presence of reducing sugars, such as fructose, glucose, lactose, and mannose, via the nonenzymatic glycation reaction (a Maillard-like reaction). The reaction can be expected during thermal food processing, particularly in dairy products such as heated milk, milk powder, and infant formula, containing an excess of lactose, in cereal-derived products such as biscuits and breakfast cereals, containing maltose, and in heat-treated fruits, e.g., pasteurized fruit juices, rich in fructose and glucose^555^. In baked model cookies, made from wheat flour fortified with folic acid and different carbohydrates, the reducing monosaccharides glucose and fructose were most effective in depleting folic acid by about 50% of its initial content, the reducing disaccharide lactose decreased folic acid by 23%, and non-reducing disaccharide sucrose did it by about 15% only at the end of baking likely due to the cleavage into glucose and fructose. Therefore, baked products should be made from sucrose rather than from glucose and fructose when a maximum of folic acid has to be retained. In particular, heated products for diabetics made from fructose or heat-treated foods, sweetened with corn syrup or high-fructose corn syrup, may contain lower amounts of folates due to glycation reaction^556^. Fructose significantly accelerated the thermal degradation of the solution of 5-methyl-THF, but glucose did not. Ascorbic acid addition to folate with fructose before heating prevented 5-methyl-THF degradation^557^. The importance of folate glycation in fruits and vegetables remains unclear, given that antioxidants, such as ascorbic acid and phenolic compounds, are inherently present. There is no data regarding fruits and vegetables on the balance between protection by antioxidants and degradation by reducing sugars. Moreover, ascorbic acid is often added to processed products. The added amount of ascorbic acid and its own degradation rate might therefore determine whether and when glycation of folates can take place^106^.

A food constituent of particular interest is folate-binding protein (FBP) occurring in milk. It possesses different affinities to various folate vitamers, with the highest for synthetic folic acid. Its binding affinity is also influenced by the pH of the environment. Like all proteins, FBP is heat-sensitive, and denaturation affects its folate binding capacity. Raw milk retains its native FBP content whereas ultra-heat treatment of milk inactivates FBP. Data on pasteurization are inconsistent. FBP is destroyed by heat beyond the temperature of 72 °C. In pasteurized milk, FBP is only partly denatured by heating, and folate remains bound to FBP. Ultra-high-temperature milk (UHT, heated for 145 °C/5 s) and yoghurt (heated for 90 °C/10 min before inoculation) lose their FBP through denaturation due to high processing temperatures. Cottage cheese and whey products contain FBP, while hard cheese contains negligible amounts, probably due to the separation of the whey proteins during manufacturing. Freezy-drying or spray-drying for the manufacture of milk powder seems to retain most of the FBP in an active state. FBP increases the stability of folates against degradation over a range of temperatures and pH conditions. On the other hand, human in vitro and in vivo studies revealed that FBP decreases the absorption of folates from the gastrointestinal tract. This effect of FBP is dose-dependent, and it also depends on the folate form. Folic acid is more affected than 5-methyl-THF owing to the different affinities of FBP for various vitamers. The bioavailability of folates from dairy products declined with increasing amounts of FBP, in order, UHT milk, fermented milk, and pasteurized milk. For example, the bioavailability of folic acid from fortified pasteurized milk was non-significantly 6-26% less relative to that of folic acid from fortified UHT milk. It may be of importance in infants when milk formulas and gruels are the main dietary source of folate. Producers of those products should consider either denaturing the FBP or replacing folic acid with 5-methyl-THF as fortificant. The effect of bovine FBP on folate absorption for adults should be negligible, since the daily intake of FBP originating from dairy products in a mixed diet is low, probably less than 10% of the total folate intake. Exceptions could be consumers with high intakes of cottage cheese and whey products which seem to be quite rich in active FBP^75,147,149,188,189,224,227,228,558,559^. The presence of FBP in plants has recently been reported^560^. The role of FBP in the stability and bioavailability of folates is still unclear and requires further research.

#### Increasing fortificant stability by encapsulation

Encapsulation may increase the stability of folic acid, commonly used for food fortification, during food processing and storage^561–566^. Folic acid encapsulated in zein fibres and nanocapsules showed resistance to thermal treatment and ultraviolet irradiation exposure in contrast to unencapsulated folic acid^567^. Folic acid incorporated in edible alginate/chitosan nanolaminates was more stable under ultraviolet light exposure than non-encapsulated folic acid^568^. The influence of processing and storage on the stability of encapsulated folic acid in apple and orange juices was studied. Folic acid encapsulated by using mesoporous silica particles was more stable, compared to free folic acid, when the apple or orange juices were sterilized, exposed to visible or ultraviolet light, and stored at 4 °C for 28 days. Thermal, light, and storage stability of free and encapsulated folic acid was much higher in orange juice, which is rich in ascorbic acid, in contrast to apple juice, likely due to the above-mentioned protective effect of ascorbic acid^546^. The stability of encapsulated folic acid (two different matrices: whey protein concentrate and resistant starch, and two encapsulation techniques: electrospraying and nanospray drying) during storage in water solution and in dry conditions under natural light and darkness was investigated. Greater encapsulation efficiency was observed for the protein-based capsules. The best results in terms of folic acid stabilization in the different conditions assayed were also obtained for the protein-based capsules, although both materials and encapsulation techniques led to improved folic acid stability^569^. Entrapment in β-lactoglobulin and lactoferrin coacervates showed good protection for folic acid against degradation during storage treatments, such as freezing and freeze-drying^570,571^. Microencapsulation of 5-methyl-THF, a mentioned less stable alternative fortificant, in pectin-alginate gel enhanced its thermal stability during extrusion processing of starch, particularly at elevated extrusion temperatures^373^. 5-methyl-THF encapsulated with modified starch used for fortification of wheat flour had higher stability than the free compound during the breadmaking, the following storage of bread slices in polyethylene bags for 3 and 7 days at room temperature, and the toasting. The losses of the fortificant were further markedly decreased when it was co-encapsulated along with sodium ascorbate, which enhances resistance of 5-methyl-THF to thermal oxidative degradation as reported above^431^. Similar results were obtained after baking and 7 days of storage in wheat bread fortified with free or microencapsulated 5-methyl-THF, with or without sodium ascorbate. Skim milk powder was used for encapsulation^430^. The binding of folic acid to proteins, such as whey protein isolate, casein, β-lactoglobulin, α-lactalbumin, and bovine serum albumin decreased folic acid losses due to photodegradation induced by ultraviolet radiation. All those proteins may be considered carrier materials suitable for folic acid delivery in functional foods^572–577^. The stability of folic acid may be improved not only by encapsulation but also by the synthesis of some derivatives. A novel derivative, 6-deoxy-6-[1-(2-amino)ethylamino)folate]-β-cyclodextrin, showed enhanced photostability against ultraviolet light compared to free folic acid and may provide a more stable source of folate as a food additive in both the solid state and aqueous solution^578^.

#### Industrial production of folate

Folic acid, which does not occur naturally in foods, is industrially produced by chemical synthesis. It is used not only in fortified foods but also in dietary supplements^1,34,98,139,222,579–612^. The pharmaceutical industry offers folic acid for therapeutic and prophylactic use. The major part, about 75%, is used for feed enrichment in animal nutrition^86,245,353,613–617^. All commercial syntheses are based on the concept of a three-component, one-pot reaction of triamino-pyrimidinone with a three-carbon compound of variable structure (e.g., halogen derivatives of propanal, propanone, and propane) and *p*-aminobenzoyl-L-glutamic acid to yield folic acid. There are some alternative approaches for the synthesis of folic acid. In a two-step procedure, 2-hydroxymalondialdehyde is firstly condensed with *p*-aminobenzoyl-L-glutamic acid, forming a diimine, which subsequently reacts with triamino-pyrimidinone to obtain folic acid. Another viable method starts from 6-formylpterin. Condensation of 6-formylpterin with the diester of *p*-aminobenzoyl-L-glutamic acid, followed by reduction of the Schiff base with sodium borohydride and hydrolysis, leads to folic acid^1,86,353,618^. The synthetic yield of folic acid is around 84%^618–624^. 5-methyl-THF, which may be used as an alternative to folic acid for food fortification and dietary supplementation, is produced synthetically from folic acid^1,353,625–627^.

Attempts have been made to develop a biotechnological method of folate production for a future switch from current chemical manufacturing to a sustainable fermentative one. Folate production capacity has been studied in various strains of the yeast *Saccharomyces cerevisiae* and yeast species isolated from environments such as marine and tropic milieus, including fruits, vegetables, fish, and insects, as well as in some bacteria^103,628^. Recently, the yeast *Scheffersomyces stipitis* has been shown to produce folate at concentrations of 3.4 mg/L under optimized cultivation conditions, the highest value obtained during fermentation in microorganisms with natural production ability^629,630^. Genetically modified folate overproducing strains of some fungi and bacteria have been constructed, e.g., *Ashbya gossypii*, *Escherichia coli*, and *Bacillus subtilis*, the first being the best folate producer reported to date with folate titers of 6.6 mg/L (i.e., 146-fold higher than the wild strain)^631–634^. However, despite the improvements in folate production by microorganisms that have been achieved, the industrial microbial production of folate is still far from being economically feasible due to very low yields. The fermentation process is not competitive with low-cost industrial chemical synthesis as yet. Thus, more efforts are needed to increase folate production levels through metabolic engineering^1,631,632,634,635^.

#### Fortification

Clinical and epidemiological data show that folate deficiency is widespread in many populations. Limited bioavailability and loss of folate during food processing and storage, and false nutrition or malnutrition, make the possibilities of reaching recommended targets for folate intake through food folates alone still rather uncertain. Fortification, the process of adding micronutrients to an appropriate food vehicle in order to correct or prevent community-wide deficiencies, has been proposed as one way to enhance folate intake. The advantage of food fortification is, compared with supplementation, that there is no need to change dietary habits^121,122,194,233,333,580,581,596,631,636–688^. Over 70 countries, including countries of North America, South America, West, East, and Southern Africa, Central and Southeast Asia, Australia, and New Zealand, have implemented mandatory folic acid fortification of foods until 2022, starting with the United States of America in 1998^680,689–692^. In Europe, only Moldova, Kosovo, and, most recently, the United Kingdom mandate fortification of food with folic acid^693^. Voluntary fortification of food products with folic acid takes place in a lot of countries (in some of them also along with the mandatory one), e.g., Canada, the U.S.A., the Dominican Republic, Sierra Leone, Sudan, Eswatini, Saudi Arabia, Kuwait, Iraq, India, Bangladesh, Myanmar, China, and many European countries^458,587,612,614,642,654,660,688,694–709^. An interesting economic analysis of possible folic acid food fortification is available from the Netherlands^710^.

The most common food vehicles for mandatory folic acid fortification are wheat flour, maize flour, and rice^328,331,333,637,653,680,689,711–724^. On a voluntary basis, foods such as breakfast cereals, cereal bars, cereal snacks, crisp bread, pasta, baby foods, biscuits, buns, cakes, pastries, milk, milk powder, dairy products, sweets, fruit juices, coffee, cocoa, soft-drinks, soy products, dried soups, margarine, fat spreads, and table salt, are fortified with folic acid^98,122,174,224,300,458,559,614,642,643,654,660,666,671,688,692,694–698,701,725–736^. Further strategies for fortification have been investigated, e.g., fortification of salt^737–743^, sugar^738^, tea^744,745^, mineral water^746,747^, and bouillon cubes^748,749^.

#### Biofortification

Biofortification refers to a strategy where conventional plant breeding techniques, genetic engineering, and agronomic interventions are used to enhance the nutrient content of food crops. Biofortification has the advantage of being more sustainable by eliminating the need to fortify each batch of food, as is the case with fortification^653,750,751^. Biofortification, i.e., the enhancement of natural folate content in plants, holds the potential to reach the required folate levels, which are low, particularly in staple crops, such as rice, potato, maize, wheat, and cassava^752–755^. Biofortification by conventional breeding relies on an inheritance of favourable quantitative trait loci from sexually compatible parental lines. It is constrained by the natural variation of the desired trait present in the available collection of crop germplasm, as well as by being time-consuming. On the other hand, conventional breeding, though limited in its potential for folate level improvement, is promising, as it might face lower regulatory restrictions compared with genetic engineering, hence allowing a more rapid implementation in agriculture, reaching the populations in need^756^. Breeding approaches focus on the pursuit of sufficient folate variation in target plant germplasm. Screening vast collections of germplasm may reveal greater diversity and thereby favour the applicability of breeding strategies^25,752,757,758^. Variation in folate levels has been analysed in different wheat^131,759–766^, barley^458,762,767^, rye^131,762,768,769^, oat^762,770^, rice^330,452,765,766,771–773^, maize^766,774,775^, foxtail millet^776,777^, potato^151,152,778,779^, lentil^63,780,781^, soybean^782–784^, faba bean^411,785^, common bean^411,781,786–788^, adzuki bean, mung bean, cowpea^411^, winged bean^529^, pea^63,411,781,789^, chickpea^63,781^, strawberry^49,74,484^, tomato^181,486,533^, pak choi^790^, lettuce^64^, spinach^45^, and coriander^523^ accessions. A lot of efforts have been made on folate biofortification in plants by genetic engineering approaches. The possible folate enhancement is not restricted by limited natural variation in the folate content of a particular plant species, as is the case of plant breeding. Genetic engineering makes use of fundamental knowledge on the complex matter of folate biosynthesis and its regulation, part of which remains to be elucidated. The main goal is to design an effective folate enhancement strategy, considering both folate accumulation and stability, adaptable to the specific metabolism of target tissues in different crops because different biosynthetic steps need to be engineered in each one to result in a substantial folate increase. Manipulation of genes encoding enzymes for, e.g., folate biosynthetic and salvage pathway, polyglutamylation, and folate binding proteins, has been carried out with some achievements^2,10,25,34,303,310,560,752,754,776,791–802^. Compared with non-genetically modified plants, folate content increased 0.17–150-fold in rice grains, 2–25-fold in tomato, 2–12-fold in potato tubers, 2.3-fold in wheat grains, 2.1–8.5-fold in lettuce, 2–4.2-fold in maize grains, 3-fold in Mexican common bean, and 1.3–4-fold in *Arabidopsis* leaves after genetic modification^532,752,754,791,803–812^. The maximum level of folate biofortification reached in rice seeds exceeds the recommended daily allowance for an adult person (400 μg) more than fourfold. Cooking experiments demonstrated around 45% folate losses after 30 min of boiling. Assuming an average bioavailability of natural folates in a mixed diet of about 50%, it is very likely that 100 g of the biofortified rice grains can satisfy the daily folate requirement for an average adult person or at least supply most of it^812^. None of the folate biofortified crops has been approved for commercial release.

In addition to biofortification, another strategy for increasing folate content in foods is its in situ production during fermentation by folate-synthesizing microorganisms. Lactic acid bacteria, e.g., lactococci, streptococci, and lactobacilli, widely used as starter cultures for the fermentation of a large variety of foods, have been intensively investigated. Most lactococci and streptococci, such as *Lactococcus lactis* and *Streptococcus thermophilus*, have the ability to synthesize folate de novo. This was already discussed above with fermented dairy products, such as yoghurt. On the other hand, many lactobacilli are not capable of producing folate de novo because some genes coding for enzymes involved in folate biosynthesis are lacking in their genome; this is the case for, e.g., *Lactobacillus acidophilus*, *Lactobacillus plantarum*, and *Lactobacillus reuteri*. However, they can often synthesize folate if some precursors, e.g., *p*-aminobenzoic acid, are available in their environment (the culture medium or food). Some lactobacilli are folate auxotrophs dependent on folate intake from exogenous sources. The ability of microbial cultures to produce or consume folate varies considerably, being a strain‐dependent trait and influenced by fermentation conditions. Proper selection of strains and their combination for starter cultures is essential to develop fermented foods with increased folate content^1,123,302,434,469,518,519,813–831^. It has been shown that different substrates, such as milk (e.g., cow and goat), legumes (e.g., soy, and white beans), cereals (e.g., wheat, oat, barley, maize, sorghum, tef, and pearl millet), pseudocereals (e.g., amaranth, chia, and quinoa), and vegetables (e.g., cabbage, beetroot, turnip, potato, oca, papalisa) are suitable to be fermented by lactic acid bacteria and hence to improve the folate content^469,819,823–825,832–843^. Similarly, bifidobacteria synthesize folate de novo or from precursors or, on the contrary, do not synthesize but utilize available folate depending on the respective strain and medium composition. Folate-producing bifidobacteria may be used for in situ fortification of fermented dairy products. For example, the addition of *Bifidobacterium bifidum* to a common yoghurt starter culture (*Streptococcus thermophilus* and *Lactobacillus delbrueckii* subsp. *bulgaricus*) brought about higher folate content in the yoghurt compared with the yoghurt obtained by fermentation of milk with the common yoghurt starter^1,146,258,517,815,817,824,828,831,844–846^. Propionibacteria, e.g., *Propionibacterium freudenreichii*, have been demonstrated as folate-producing bacteria. High differences in folate production can be found between different strains. The advantage of propionibacteria is their ability to synthesize vitamin B_12_ as well. For example, co-inoculation of kefir grains, used to prepare kefir, with a folate and vitamin B_12_ producing *Propionibacterium freudenreichii* strain resulted in an increased content of both important vitamins in kefir. Similarly, the co-cultivation of a folate producer, *Lactobacillus plantarum*, with a vitamin B_12_ producer, *Propionibacterium freudenreichii*, in a whey permeate medium led to a natural dietary supplement with an optimal ratio of folate and vitamin B_12_^146,847–852^. Yeasts, e.g., *Saccharomyces cerevisiae* and *Candida glabrata*, have a high capability of producing folate. Careful selection of strain opens possibilities for optimizing folate content in yeast-fermented foods, such as bread and African types of porridges^103,835,838,853–855^. A combination of the lactic acid bacteria and yeasts may be useful in increasing folate through fermentation, e.g., in pearl millet fermented gruel (*Lactobacillus fermentum* and *Pichia kudriavzevii*)^856^, in ogi, a fermented maize gruel, (*Lactobaccilus plantarum* and *Candida tropicalis*)^841^, and in idli, a steamed cake based on fermented mixture of rice and black gram, (*Lactococcus lactis* and *Saccharomyces boulardii*)^857^. In the case of idli, levels not only of folate but also of riboflavin were enhanced^857^.

Searching for native strains of folate-producing microbes from different niches (ethnic foods, fruits, vegetables, cereals, vegetation, animals, soil, and so on) is very important for the development of novel in situ fortified products^103,146,258,818,827,832,834,837,843,847,852,853,855,856,858–865^. For example, folate levels were 3–5-fold higher in white wheat bread leavened with a *Saccharomyces cerevisiae* strain, originally isolated from the Rainbow trout intestine, compared to white wheat bread leavened with a commercial Baker’s yeast strain^866^. A yoghurt with a starter culture consisting of *Streptococcus thermophilus*, a high folate producer, and *Lactobacillus delbrueckii* subsp. *bulgaricus*, a folate consumer, usually contains 2-fold higher amounts of folate compared to the original milk. A new *Lactobacillus delbrueckii* subsp. *bulgaricus* strain, capable of producing folate, was isolated from artisanal yoghurts of the northwestern region of Argentina. The fermentation of milk with a starter composed of two folate producing *Streptococcus thermophilus* strains and a folate producing *Lactobacillus delbrueckii* subsp. *bulgaricus* strain resulted in the yoghurt containing 4.5-fold more folate compared with the non-fermented milk^826,861^. Another way to obtain folate high producing microorganisms for in situ fortification is genetic modification. Genetic engineering was successfully used to increase folate production by *Lactococcus lactis* and to transform a folate consumer, *Lactobacillus gasseri*, into a folate producer^867–872^. Use of genetically modified wine yeasts for wine fermentation resulted in elevated folate content in wine^516,873^. However, despite the approach being efficacious in improving folate levels, the selection of natural overproducers has not gained favour due to the legislative limitations and negative perception of genetic modification by consumers^1,814,835^.

The folate content of in situ fortified fermented food products enriched by folate-producing microorganisms is still low. Such products would provide economic benefits to food manufacturers since increased “natural” folate concentrations would be an important value-added effect without increasing production costs. Consumers would benefit from such products since they could increase their folate intake while consuming products that form part of their normal diet^1,146,814,842,874^.

## Kinetics in humans and homeostasis

### Compartmentalization

Different forms of folate have different abilities to be transported through the biological membranes, which results in compartmentalization of folate between extracellular space, cytosol, and mitochondria, with smaller folate amounts located in the nucleus^875^. The most important determinant of this compartmentalization is the polyglutamate tail. Monoglutamate, but not polyglutamate forms are substrates to several folate-transporting proteins and are able to cross plasma membranes^275,876^ or mitochondrial membranes^877,878^. Without being metabolized, polyglutamate forms are therefore not absorbed from the intestine or are trapped within the intracellular compartment where the polyglutamate tail is added^879^. Another factor contributing to this compartmentalization is the presence of the one-carbon units. Folates with bound carbon units (i.e., methyl-THF, methylene-THF, formyl-THF) are unable to cross the mitochondrial membrane^880^.

### Folate transporters

Folate molecules have hydrophilic properties and passive diffusion across cell membranes is minimal. Specific transport proteins are required to mediate folate transfer across cell membranes, either during intestinal absorption or distribution into tissues. Several folate transporters have been identified and characterized: the reduced folate carrier, the folate receptors, and the proton-coupled folate transporter^881^. Reduced folate carrier is expressed ubiquitously in all tissues, but it is selective only for the reduced folate forms^882,883^. Proton-coupled folate transporter seems to be the major transporter at low pH levels and in intestine^884^. Both reduced folate carrier and proton-coupled folate transporter are the most common ways used by folates to reach tissues. The membrane-bound folate receptor has the highest affinity for folate (Kd ∼0.1–1 nmol/l), characteristically binds folic acid, reduced folates, many antifolate drugs, and folate conjugates, and transports them by a non-classical endocytic mechanism^881,885^. Furthermore, folates are substrates for less-selective transport proteins like organic anion transporter OATP1B1, multidrug resistance-associated proteins (MRPs), and the breast cancer resistance protein^275,886^.

### Absorption

The most common form of folate used in nutrient supplementation is folic acid, even though it is not normally present in food or in nature as aforementioned. Folic acid has high bioavailability, it is stable, and can be quickly converted into the active tetrahydrofolate forms. Dietary folate exists in polyglutamate form which must be converted into monoglutamate before absorption. This reaction is catalysed by the enzyme folate conjugase on the brush border of enterocytes in the proximal small intestine^887,888^. Along the brush border folate conjugase, enterocytes express intracellular folate conjugase, an enzyme with similar activity but different properties^888,889^. The absorption of monoglutamate forms occurs mostly by a saturable mechanism via the reduced folate carrier, folate receptor, and especially proton-coupled folate transporter with Km of 1–3 μM^881,890,891^. The pH optimum for the active saturable transport is 5.5–6.0, which explains why antacids seem to reduce folate absorption. An additional passive, unsaturable absorption pathway exists that is used when the intraintestinal folate concentrations exceed 10 μM^209^.

Oxidized and reduced forms of folate are absorbed to a similar degree; however, the reported bioavailability values range from 10 to 98%^192,199,211^. 5-methyl-THF is absorbed unchanged. Other forms, including 5-formyl-THF, are converted to 5-methyl-THF by intestinal mucosa (as the organ most responsible for adding the methyl group and reducing the vitamin) or in a small degree by the liver^887,892^.

#### Improving folate bioavailability through processing

Because intestinal deconjugation of polyglutamates to monoglutamates is the rate-limiting step in intestinal folate absorption, an increase of folate monoglutamate portion in foods may improve the bioavailability of dietary folate^1,37,216^. The tissue and cell disruption during processing (e.g., mixing, cutting, crushing, freezing/thawing, and high pressure treatment) makes native polyglutamyl folates accessible for endogenous conjugases (γ-glutamyl hydrolases) and results in the hydrolysis to monoglutamyl folates in vegetables (e.g., leeks, cauliflower, broccoli, spinach, soybeans, green beans, cowpea leaves, turnip, and carrot) and fruits (e.g., orange, papaya, sweet cherry, strawberry, and blackberry). Deconjugation could be affected by several factors, such as differences in native conjugase activity, the presence of endogenous conjugase inhibitors, and the use of heat during processing. Heating, e.g., during blanching and steaming, largely inactivates conjugases such that long-chain polyglutamyl folates are preserved. Food processing may by itself increase folate bioavailability. On the other hand, higher losses of total folate after treatment may occur because of leakage due to matrix disruption and oxidative degradation of monoglutamate forms due to their lower stability^37,75,117,180,234,394,399,403,414,473–475,477,480,533,893^. Changes in folate glutamylation profiles during maturation were observed in cowpea leaves, winged beans, tomato, avocado, banana, and papaya^38,399,529,893^.

### Distribution and tissue retention

After absorption, the monoglutamate folates are distributed to tissues and converted to the polyglutamate form by the enzyme folylpolyglutamate synthetase. The majority of folate entering tissue cells is in the form of 5-methyl-THF monoglutamate or is quickly metabolized to this form, which has a low affinity for folylpolyglutamate synthetase^894^. For the polyglutamylation to be effective and to achieve tissue retention, 5-methyl-THF needs to be first metabolized to THF via the methionine cycle. However, the passage of 5-methyl-THF through the methionine cycle is limited, especially in situations with high intracellular 5-methyl-THF levels. Under such conditions, the newly absorbed folate is not retained by the tissue and is released back into the systemic circulation, mostly as 5-methyl-THF.

The largest pool of folates is in the liver which can accumulate 50% of the total body folate content^895,896^. Folates in the liver may be directed into three metabolic pathways. 1) The folate monoglutamates can be converted to polyglutamate forms to be retained; 2) these polyglutamate stores can be hydrolysed to monoglutamates by the enzyme glutamate carboxypeptidase II and released to meet the body’s requirements; and 3) the folate monoglutamates can be partially secreted into bile and excreted to the duodenum and small intestine, undergoing enterohepatic folate circulation^897^. After the liver, the pancreas is the second largest store of folate^898^.

### Elimination and excretion

Any folate excess not retained by tissues is excreted in the urine and faeces, in an intact form or as metabolites. Daily excretion of folates in humans is estimated to be <1% of the total folate body pool. Only about 5% of ingested folate is excreted with urine in unchanged form at physiological doses^899^. The mechanism of folate breakdown is incompletely understood but happens in most tissues and primarily involves the irreversible oxidative cleavage of the C9-N10 bond, forming various pterins and folate-derived amines (*p*-aminobenzoyl-polyglutamates). The pterin moiety is excreted in bile and appears in faeces. Faeces usually contain high concentrations of folates, but most of them originate from the bacterial synthesis in the lower gut. The *p*-aminobenzoyl-polyglutamates are further hydrolysed to monoglutamates by lysosomal glutamylhydrolase and acetylated, forming the main metabolites *p*-aminobenzoylglutamate and its acetylated form, *p*-acetamidobenzoylglutamate^900^. This metabolic pathway is present in all tissues, with the highest activity being detected in liver and kidney ^901^. *p*-Acetamidobenzoylglutamate and *p*-aminobenzoylglutamate are subsequently excreted in urine.

### Enterohepatic circulation

Folates are subject to enterohepatic circulation which limits the loss of the pterin moiety in faeces. After excretion in bile, the folates are rapidly reabsorbed for redistribution to the liver and tissues. The importance of this process has been demonstrated in animal studies^897^ which have shown that bile drainage leads to the rapid decrease in folate serum concentrations by 60–70% within 6 h. The enterohepatic cycle seems to play a significant role in maintaining folate homeostasis and its interruption may affect folate availability more than dietary deficiency.

## Physiological function

Even though folates are distributed and enter cells as monoglutamates, the functional cofactors are in the form of polyglutamates. The polyglutamate tail not only helps with vitamin retention in cells but also increases the folate affinity for folate-dependent enzymes by as much as 1000-fold^275,902^.

Folate coenzymes are involved in three major metabolic cycles: the purine cycle, the thymidylate cycle, and the methionine cycle. The folates enter these cycles with the bound one-carbon unit in different oxidation states (5-methyl-THF, 5,10-methylene-THF, 10-formyl-THF) which are cleaved off during biochemical reactions. The cofactors then exit the cycles as dihydrofolate or THF. The central pathway interconnecting these different biochemical functions of folates is the regeneration of the one-carbon units to THF – the one-carbon folate metabolism or cycle.

### One-carbon folate cycle

**Fig. 2:**
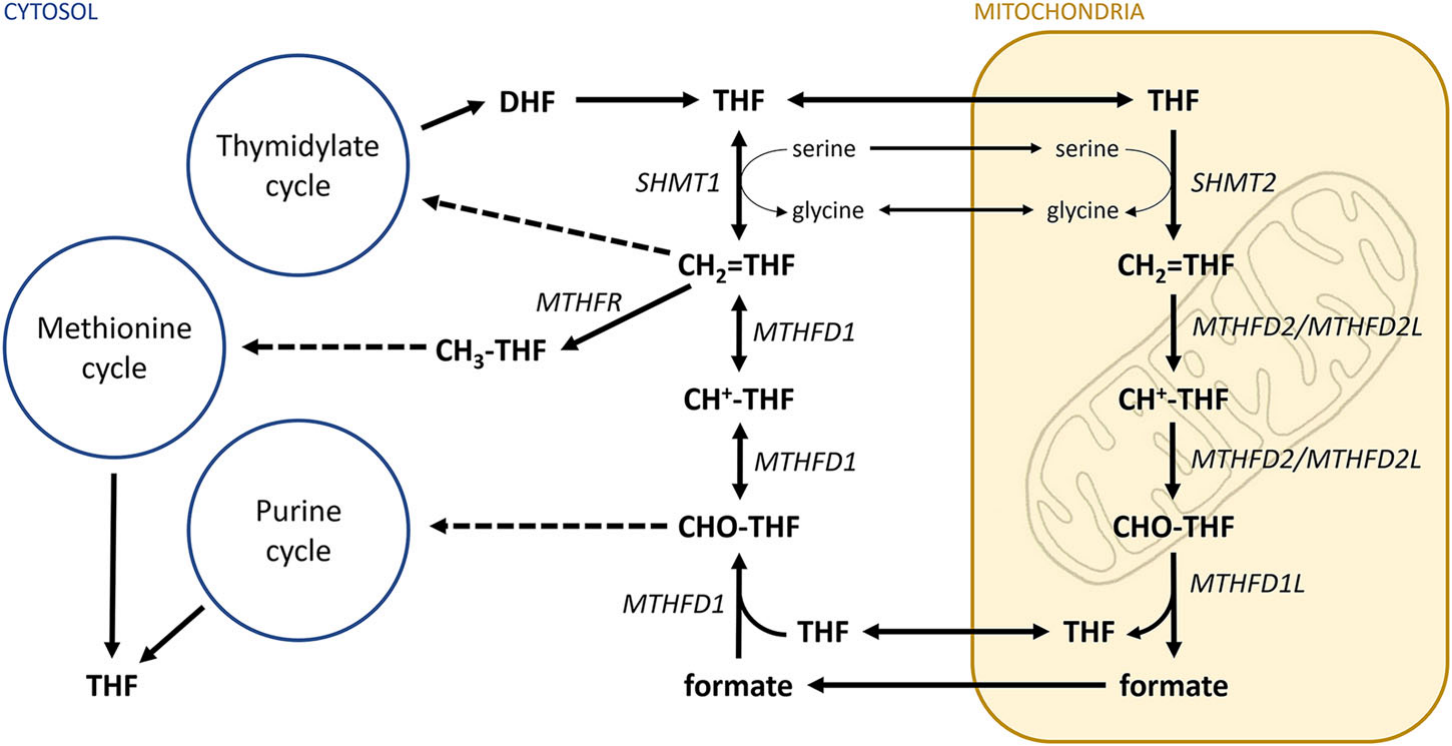
The one-carbon folate cycle. In the mitochondrial compartment, one-carbon units are attached to tetrahydrofolate, oxidized, and transported into cytoplasm, where they are available for the three principal folate biochemical pathways. See text for detailed description. DHF dihydrofolate, THF tetrahydrofolate, SHMT serine hydroxymethyltransferase, CH_3_-THF 5-methyl-THF, CH_2_=THF 5,10-methylene-THF, CHO-THF 10-formyl-THF, CH^+^-THF methenyl-THF, MTHFR methylenetetrahydrofolate reductase, MTHFD1 methylenetetrahydrofolate dehydrogenase 1, MTHFD1L methylenetetrahydrofolate dehydrogenase 1 like, MTHFD2 methylenetetrahydrofolate dehydrogenase 2, MTHFD2L methylenetetrahydrofolate dehydrogenase 2 like.

The main source of one-carbon units for folate-mediated methylation in humans is serine. In a reversible reaction catalysed by serine hydroxymethyltransferase (SHMT), the serine β-carbon is transferred to tetrahydrofolate to form methylene-THF. Mammals contain two distinct isoforms of SHMT encoded by different genes: cytosolic SHMT1 and mitochondrial SHMT2^903^. It has been shown in healthy volunteers^904^, in cell culture^905^ and in most cancer cells^906^ that the majority of one-carbon units transferred to methionine originates in the mitochondria (i.e., SHMT2). Thus, the one-carbon folate cycle may be thought to start with the mitochondrial SHMT2-catalysed demethylation of serine in the presence of tetrahydrofolate to produce glycine and methylene-THF (Fig. 2). Next stage is the two-step oxidation of methylene-THF to methenyl-THF (CH^+^-THF) and 10-formyl-THF. These oxidative reactions are catalysed by isoenzymes methylenetetrahydrofolate dehydrogenase 2 (MTHFD2) or MTHFD2-like (MTHFD2L) and consume oxidized nicotinamide adenine dinucleotide (NAD^+^), which is reduced NADH. The final mitochondrial step is the cleavage of 10-formyl-THF into formate and free THF by the enzyme methylenetetrahydrofolate dehydrogenase 1 like (MTHFD1L). Formate is a critical intermediate metabolite. Along with free THF, it is able to cross the mitochondrial membrane and its availability determines the direction of the reversible cytosolic pathway^907^.

In the cytosol, the formate is incorporated back into tetrahydrofolate to form 10-formyl-THF (catalysed by methylenetetrahydrofolate dehydrogenase 1; MTHFD1). 10-formyl-THF is required for the de novo purine synthesis in the purine cycle. Alternatively, 10-formyl-THF can be sequentially reduced to methylene-THF by MTHFD1. The further role of methylene-THF depends on the cellular demands: it can complete the folate cycle by remethylation of glycin to serin by SHMT1; it can enter the thymidylate cycle to generate dTMP from dUMP; or it can undergo final reduction to 5-methyl-THF by methylenetetrahydrofolate reductase (MTHFR) and enters the methionine cycle. It is important to note that this last reduction is physiologically irreversible, and the methionine cycle is the only pathway that utilizes 5-methyl-THF. Disturbance of the methionine cycle, especially of the first enzymatic step catalysed by methionine synthase, can lead to folates being trapped in the 5-methyl-THF form and the inability of the cell to produce purines and dTMP.

The folate compartmentalization leads to the separation of cytosolic and mitochondrial one-carbon metabolic pathways which remain connected only through specific metabolites. Even though the majority of one-carbon demand is in the cytosol, almost all THF substrates to meet this demand are produced in the mitochondria. Why such a parallel set of biochemical pathways exists is not clear. However, it has been shown that the mitochondrial 10-formyl-THF production accounts for almost 50% of NAD^+^/NADP^+^ consumption and NADH/NADPH production in cell^880^. One-carbon oxidation might be localized in mitochondria to uncouple it from glycolytic and other metabolic reactions in the cytosol, which might be disrupted by NAD^+^ depletion that would happen if one-carbon oxidation should take place in cytosol^359,877^.

### Nucleotide synthesis – thymidylate and purine cycles

Human cells produce purines in the cytosol both via salvage and de novo biosynthesis pathways. The de novo synthetic pathways consist of 10 steps which convert phosphoribosyl pyrophosphate into inosine monophosphate and further to adenosine and guanosine^908^. The conversion into inosine monophosphate requires two one-carbon units from 10-formyl-THF, which are incorporated at the 2’- and 8’-positions of the purine ring. These reactions release free THF and are catalysed by glycinamide ribonucleotide transformylase (GARTF) and 5-amino-4-imidazolecarboxamide ribonucleotide (AICAR) transformylase^909^.

Methylene-THF is also required for the synthesis of DNA, specifically for the production of dTMP. Enzyme thymidylate synthase catalyses the transfer of one carbon from methylene-THF to the 5’-position of dUMP. The THF molecule also serves as the reducing agent to reduce this one-carbon to methyl group to form dTMP. THF is oxidized to dihydrofolate and needs to be reduced back to THF before it can be reutilized in the folate cycle. This THF regeneration step is catalysed by dihydrofolate reductase. Significant thymidylate synthase activity was detected only in replicating, especially fast-growing cells, and thymidylate synthase activity and protein levels are subject to cell cycle variations, associated with the onset of the S-phase^910^. Some evidence suggests that thymidylate synthase is active only as part of the replitase complex in the nucleus (see below) and not in cytosol^911^. Dihydrofolate reductase inhibiting drugs (e.g., methotrexate and others, see later) are therefore selective inhibitors of rapidly growing cells and have proven to be effective antineoplastic agents^912,913^.

### Methionine cycle

5-methyl-THF is a substrate for methionine synthase (one of only two vitamin B_12_-dependent enzymes in mammals) and the methionine cycle^914,915^. 5-methyl-THF acts as a methyl donor for homocysteine methylation, forming methionine and free THF. Methionine can be further metabolized to S-adenosylmethionine, which acts as a methyl donor for a broad spectrum of reactions, including methylation of histones, DNA, neurotransmitters, phospholipids, and synthesis of glutathione, phosphotidylcholine, and creatine. These reactions are critical for the regulation of gene expression, development, and genomic stability (the role of vitamin B_9_ and B_12_ in genomic stability is complexly reviewed in ref. ^916^). The methionine cycle is very sensitive to inadequate folate levels. When folate concentrations are low, the remethylation of intracellular homocysteine is disrupted which leads to increased plasmatic homocysteine concentrations. Total plasma homocysteine can be considered an indirect indicator of folate insufficiency^917,918^ but it should be stressed that hyperhomocysteinemia can be mediated by other causes as well^919^.

These cytosolic pathways show different relative importance in various cell types. In quickly proliferating cells, such as stem cells, hematopoietic cells, or cancer cells, the majority of one-carbon units are incorporated into nucleotides (purines and dTMP)^877,920^. In slowly proliferating cells (such as cultured fibroblasts) most of one-carbon flows through thymidylate and methionine cycle^921^. In non-proliferating cells, however, one-carbon groups almost exclusively enter the methionine cycle. In the liver and kidney – tissues with the highest activity of one-carbon anabolic reactions – S-adenosylmethionine is required for the synthesis of creatine, which accounts for almost 80% of all methylation reactions in the body^922^.

### Nuclear folate metabolism

**Fig. 3:**
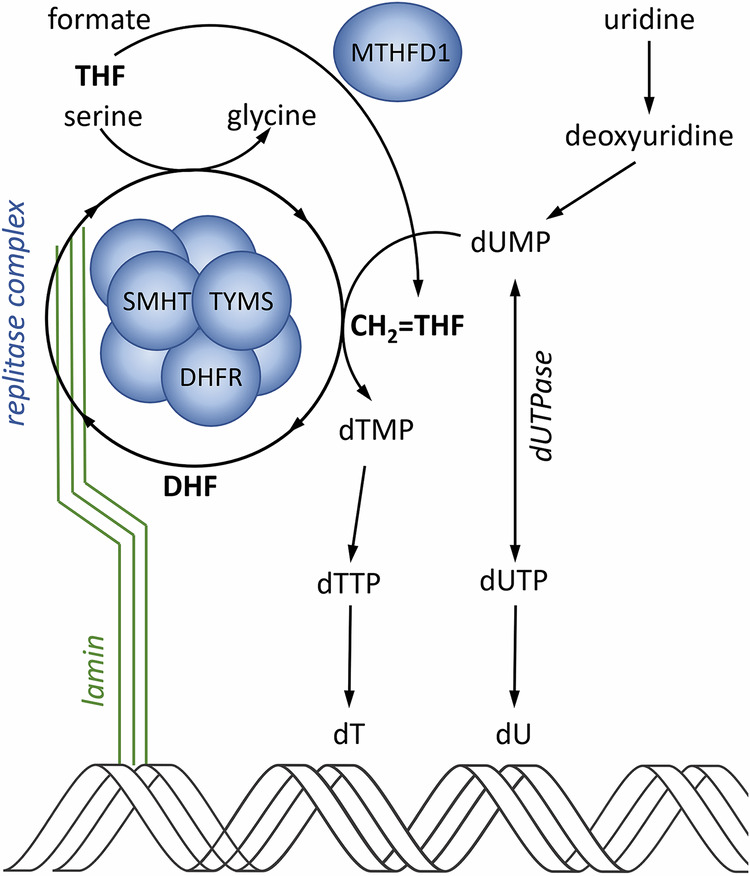
Nuclear multienzyme complex and thymidylate cycle. SUMOlyated cytosolic enzymes are transported into the nucleus where they form a multienzyme complex (replitase), responsible for local production of dTMP required for DNA synthesis. CH_2_=THF 5,10-methylene-THF, DHF dihydrofolate, THF tetrahydrofolate, DHFR dihydrofolate reductase, dT deoxythymidine, dTMP deoxythymidine monophosphate, dTTP deoxythymidine triphosphate, dU deoxyuridine, dUMP deoxyuridine monophosphate, dUTP deoxyuridine triphosphate, MTHFD1 methylenetetrahydrofolate dehydrogenase 1, SMHT serine hydroxymethyltransferase, TYMS thymidylate synthase.

Most of the folate one-carbon cycle with connected biochemical pathways occurs in mitochondria and cytosol, however, evidence suggests that at least some of these reactions are functional in the nucleus as well. In the nuclei of S-phase cells, but not during the G1-phase, a multienzyme complex was detected which contains enzymes required for the salvage pathway, de novo synthesis of dTMP, and DNA replication^923^. This complex was termed replicase. It is localized to the sites of DNA replication *via* nuclear lamina anchors^924^. The formation of replicase starts in the S-phase with increased activity of small ubiquitin-like modifier (SUMO) ligase, SUMOylation of cytosolic SHMT1, thymidylate synthase, dihydrofolate reductase and MTHFD1, and the translocation of these enzymes into the nucleus. There, these SUMOylated proteins directly bind to other nuclear enzymes and form an active functional replicase complex. Both serine (via SHMT1 or nuclear isoform SHMT2α) and formate (via MTHFD1) serve as one-carbon donors for the production of methylene-THF, which enters the nuclear thymidylate cycle to provide dTMP for DNA synthesis (Fig. 3). Impaired nuclear folate metabolism leads to suppression of dTMP production and increases dUTP production and incorporation into DNA. Such uracil misincorporation may induce DNA breaks^925,926^. It has been suggested that DNA uracil levels could present a biomarker for insufficient folate status^925^, however, a significant inverse correlation was detected only for vitamin B_12_ status^927^.

There is no strong evidence that either folate-dependent de novo purine biosynthesis or methionine cycle exists in the nucleus. Enzymes required for de novo purine biosynthesis localize exclusively to the cytosol^928,929^. Likewise, homocysteine remethylation to methionine also appears to be a cytosolic process, as the enzymes that compose this cycle are exclusively cytosolic^930–932^.

## Folate deficiency, folate supplementation and claimed risks

### Folate deficiency and related disorders

Folate deficiency can be caused by various factors. The most common are dietary insufficiency or malabsorption. Deficient absorption of folates is present in many pathological conditions, e.g., in alcoholism, celiac disease, amyloidosis, short bowel syndrome, or gastric bypass^933^. Elevated pH, which occurs in achlorhydria, or as aforementioned after extensive use of antacids, can also decrease folate absorption. Many drugs (e.g., methotrexate, phenytoin, trimethoprim, and possibly sulfasalazine) can antagonize folate utilization or inhibit its conversion to active forms^934,935^. Situations with increased requirements for folates (typically in pregnancy) can also cause symptoms of folate deficiency. Furthermore, genetic polymorphisms or mutations of the proteins involved in one-carbon metabolism (e.g., MTHFR) or folate uptake (e.g., reduced folate carrier) also increase the risk of folate-associated diseases^936^.

Given the broad functional importance of folate-connected metabolism in the body, it is unsurprising that the symptoms and dysfunctions caused by folate deficiency vary considerably. A “classical” symptom is megaloblastic anaemia: the formation of large abnormal nucleated erythrocytes, caused by inhibited maturation of erythropoietic precursor cells. Hypersegmented neutrophils are also pathognomonic of the disease. Poor folate status can cause abnormalities in DNA synthesis, protein synthesis and posttranslational modifications, and in gene expression, which may result in chronic diseases such as cardiovascular diseases, neuropsychiatric disorders (cognitive dysfunction, depression, psychosis, memory impairment), or cancer. Several cancers have been associated with folate deficiency, such as colorectal, prostate, and breast cancer^937^. The causes of carcinogenesis in low folate status are hypothesised to be the uracil misincorporation^938^ and hypomethylation of DNA causing dysregulated gene expression^916,934^.

The methods for folate detection are summarized in Table 2 with details in the Supplementary Table S1.

**Table 2:** Overview of analytical techniques for detection of vitamin B_9_

Technique	Sensitivity nmol/L	Analytes	Matrix	Advantages	Disadvantages	Ref.	Publication years
LC-MS	LLOQ -LOQ 0.03–1.63 × 10^3^ (B_9_-p-ABGA – B_9_)	B_9,_ B_9_-THF, B_9_-MTHF, B_9_-FTHF, and B_9_-p-ABGA (including B_1,2,3,5,6,7,12,_and its vitamers)	* whole blood * plasma * milk * tears * serum * mice brain * mice plasma * mice tissue	* short analysis time with multiple analytes * small sample volume (50–250 µL) * simple sample preparation * MRM * small solvents volumes	* complicated sample preparation (breast milk) * complicated gradient elution in some methods * SIM in some methods * not sufficient validation parameters * long analysis time in some method	^1248–1256^	2015–2024
HPLC-PDA	LOD 0.39 – 407.79 (B_9_-MTHF – B_9_)	B_9_ and B_9_-MTHF (including B_2,12_)	* urine * serum	* simple sample preparation * rapid	* synthesis of nanocomposite for sample preparation in some method * not sufficient validation parameters for all analytes	^1257,1258^	2021–2022
Sensors/nanodots/CL/FLD/ECD	LOD B_9_ 0.28–1 × 10^3^	B_9_	* serum * plasma * urine * artificial cerebrospinal fluids	* simple sample preparation * small sample volume (10 µL) * cheap * simple * selective * no need of organic solvents	* research only - not commercially available * 1 mL serum in some methods * synthesis of materials * sensor or electrode prepared in laboratory	^1259–1273^	2017–2024
Microbiological test kits	LLOQ B_9_ 6.8	B_9_	* serum	* small sample volume (100 µL)	* high price (working in duplicate recommended) * long analysis time (24 h)	^1274^	2024
ELISA kits	LOD B_9_ 62.08 × 10^−3^–0.05	B_9_	* serum * plasma * tissue * cell * cell culture supernatant * cell lysates * others	* small sample volume (50–250 µL) * one kit for various matrices * sensitive	* for research only * cross reactivity with analogues * time and money consuming for small sample series	^1275–1277^	2021–2024

*LOD* Limit of Detection, *LOQ* Limit of Quantification, *LLOQ* Lower Limit of Quantification, *B*_*1*_ Thiamine, *B*_*2*_ riboflavin, *B*_*3*_ Niacinamide, *B*_*5*_ pantothenic acid, *B*_*6*_ *pyridoxine* B_6_-PM pyridoxamine, *B*_*6*_*-PA pyridoxic acid* B_7_ biotin, *B*_*9*_ *folic acid* B_9_-p-ABGA para-aminobenzoyl glutamic acid, *B*_*9*_*-THF* tetrahydrofolic acid, *B*_*9*_*-FTHF* 5-formyltetrahydrofolate, *B*_*9*_*-MTHF* 5-methyltetrahydrofolate, *B*_*12*_ cyanocobalamin, *CL* Chemiluminescence, *ECD* Electrochemical Detection, *FLD* Fluorescence Detection, *LC-MS* Coupling of Liquid Chromatography and Mass Spectrometry, *MRM* Multiple Reaction Monitoring, *SIM* Selected Ion Monitoring.

### Pregnancy, current opinions on folate fortifications with claimed risks

Neural tube defects are relatively common congenital abnormalities with complex but incompletely understood aetiology, which include anencephaly, encephalocele, and spina bifida. Global prevalence of live births ranges from 0.8/1000 (USA), 1/1000 (EU) to 5/1000 (China)^939–941^. Folic acid supplementation has been shown to reduce the risk of neural tube defects^942,943^; however, the mechanism by which folate reduces this risk remains obscure.

Achieving optimal folate status is challenging, however, in Europe, the recommendations of folic acid supplementation during the periconceptional period (400 μg/day folic acid from preconception until the end of the first trimester of pregnancy) have been largely ineffective in reducing the neural tube defects incidence^944^. The reason seems to be poor compliance of women who start taking folic acid only after the period of neural tube closure (day 17–28 post-fertilization), as many pregnancies may go unnoticed during this timeframe.

Mandatory folic acid fortification is an effective intervention to reduce the prevalence of neural tube defects (spina bifida, anencephaly, and encephalocele)^128,585,596,599,657,670,680,682,684,685,690,691,725,726,944–993^. In the absence of population-wide fortification and given the generally poor compliance with current folic acid recommendations, optimising the folate status of mothers in very early pregnancy for protection against neural tube defects remains challenging^679,691,994,995^. Optimal folate status also has possible preventative roles in, e.g., cardiovascular disease, in particular stroke, several types of cancer, and age-related cognitive impairment^77,128,233,300,671,680,916,944,995–1028^.

Many countries have not implemented mandatory folic acid fortification owing to concerns about potential harmful effects that might be caused by the increased intake of folic acid from fortified foods^90,128,300,614,679,688,702,986,989,990,1028–1045^. Worldwide, countries with mandatory policies of folate food fortification have reported significant reductions (by 27–50%) in neural tube defects^951,965,1046^. European countries have been reluctant to introduce mandatory folate food fortification. There is a large body of literature with observational studies, clinical trials, meta-analyses, reviews, hypotheses, and speculations on the potential association between folic acid and adverse health outcomes. The main issues are 1. masking of vitamin B_12_ deficiency primarily in the elderly^674,677,680,683,967,973,1047–1052^; 2. a risk of cognitive impairment in elderly individuals with suboptimal vitamin B_12_ status^98,678,680,690,973,995,1030,1047,1053–1071^; 3. a risk of cancer^98,581,979,1017,1025,1072–1088^ with special attention to colorectal cancer^581,680,987,1006,1014,1015,1024,1086,1089–1116^, breast cancer^581,1078,1117–1121^, and prostate cancer^581,1078,1122–1127^; 4. negative health outcomes in offspring, such as hypersensitivity-related outcomes (e.g., asthma and eczema)^604,986,1128–1141^, autism^1142–1147^, child neurocognitive development^1019,1148–1151^, and others^128,603,605,606,609,680,1023,1152–1156^; and 5. presence of unmetabolized folic acid in the circulation^98,600,670,677,972,978,1036,1047,1105,1145,1157–1168^. The concerns have arisen mostly from high-dose supplementation studies that have claimed to link folic acid supplementation and adverse effects. However, there is comparatively less data on the effects that can be specifically attributed to food fortification due to numerous potential confounding factors^691^. At present, given the heterogeneity and inconsistency in the findings among studies, there is an insufficient body of evidence to support human adverse health outcomes that are a result of high amounts of folate or folic acid intake^1031,1043^. Unequivocal and credible evidence to support the purported associations is lacking^246,983,987,995,1015^.

Regarding masking of vitamin B_12_ deficiency, it refers to the fact that both folate and vitamin B_12_ deficiency gives rise to the same type of anaemia, and, in the 1940s, before the recognition that vitamin B_12_ is a cause of pernicious anaemia, folic acid used at high doses (≥5 mg) for its treatment restored normal blood values, but did not prevent the vitamin B_12_ deficiency related neuropathy, which remained progressive and could lead to irreversible neurological damage without treatment with vitamin B_12_, and so masked vitamin B_12_ deficiency and delayed its diagnosis. Folate and vitamin B_12_ deficiencies could not be diagnostically distinguished based on haematological symptoms at that time^233,690,1169^. However, current medical practice does not rely on the presence of anaemia for the diagnosis of vitamin B_12_ deficiency, which frequently presents without anaemia^614,973,986,1007,1033,1035,1052,1061^. It is estimated that it happens in about 30% of patients^973,1039,1049,1055^. Today, blood levels of vitamin B_12_ and related metabolites are directly measured as a first-line test^599,691,1035,1039,1055^. The experience of mandatory fortification of foods with folic acid in the US showed no evidence of a higher prevalence of vitamin B_12_ deficiency in the absence of anaemia or macrocytosis^128,671,988,1052^. To address the issue of masking, based on case reports from the 1940s and 1950s, a tolerable upper intake level for folic acid from fortified foods or supplements was later set as 1 mg per day, an amount which would not mask haematological signs of vitamin B_12_ deficiency^98,991,1035,1036,1039,1055,1169–1171^. Therefore, the risk of masking vitamin B_12_ deficiency and delaying vitamin B_12_ deficiency diagnosis resulting from mandatory folic acid fortification is considered unlikely^233,581,671,678,687,691,990,1039,1169,1172^.

As for cognitive impairment due to folic acid in elderly individuals with suboptimal vitamin B_12_ status, the paucity of clear data provides insufficient evidence of an increased risk of causing or accelerating cognitive impairment resulting from vitamin B_12_ deficiency^98,246,1173^. Folic acid had no significant effect on the cognitive decline of older individuals^671,691,988,991,1033,1035,1066,1169^. Considering the potential harmful health impacts, if there are, of high folic acid and low vitamin B_12_ intake, suggestions have been made to include both folic acid and vitamin B_12_ in food fortification policies. That could prevent potential adverse outcomes of imbalance of both vitamins and address a public health issue of vitamin B_12_ deficiency, widespread in all age groups, particularly among the elderly^128,333,614,637,677,690,944,953,982,1030,1051,1061,1174–1176^. In addition, vitamin B_12_ deficiency itself may be a risk factor for neural tube defects. Adding vitamin B_12_ to folic acid might further reduce the risk of neural tube defects^1059,1061,1062,1177,1178^. Fortification with both vitamins would increase the benefits and reduce the risks, but more evidence on efficacy, dosage, and feasibility is required before this could be considered^128,678,1174^.

Concerning the relationship between folic acid and the risk of cancer, the incidence of several common cancers (e.g., colorectal, breast, and prostate cancer) and total cancer in the US, Canada, and Australia has mostly remained stable or decreased since the introduction of mandatory fortification^685,691,1077,1179^. A large meta-analysis of data on 50,000 patients showed that folic acid supplementation does not significantly increase the incidence of site-specific cancer^1077^.

The Australian Health Ministers’ Advisory Council (AHMAC) found that meta-analyses of randomized control trials for colorectal, prostate, other cancer sites, and total cancer consistently demonstrated no increase in cancer risk associated with supplementation at a population level^685,691^. The European Food Safety Authority (EFSA) review similarly found no consistent association of folate or folic acid with cancer risk, and it noted that potentially adverse effects tended to manifest at intake levels in excess of the tolerable upper intake level of 1 mg daily^98^. Currently, an expert panel for the EFSA concluded that meta-analyses indicated no association between folate and colorectal cancer. Evidence from intervention studies on the relationship between folic acid supplementation and the risk of adenomas is mixed from protective effects over the null association to elevated risk. Too few studies with mixed results prevented any clear conclusion on total folate intake and risk of prostate cancer^246^. The Scientific Advisory Committee on Nutrition (SACN) summarized that findings from the different study types are inconsistent, and the evidence is inconclusive but overall does not suggest an adverse association. Meta-analyses of randomized control trials reported no effect of folic acid supplementation on colorectal cancer risk. Meta-analyses of observational studies are heterogeneous but suggested a protective association of folate intakes above about 400 µg/day. Observational studies of serum or plasma folate concentration provide no clear evidence of an association with colorectal cancer risk. Findings do not suggest a detrimental effect of folic acid/folate on overall cancer risk. Meta-analyses of randomized control trials of folic acid supplementation show no effect of folic acid on prostate cancer risk. Genetic studies suggested that higher blood folate concentrations are associated with an increased risk of prostate cancer^988^. According to an expert panel for the US National Toxicology Program (NTP), inadequate dietary folate intake increases colorectal cancer risk in humans, but there is no benefit for cancer reduction from supplements among people whose baseline folate status is adequate. There is suggestive evidence that folic acid has an adverse effect on the development of prostate cancer. Such data coming from human studies justify the need for further research^1173^. The Prime Minister’s Chief Science Advisor stated that findings from genetic studies suggested that higher blood folate is weakly associated with increased risks of colorectal and prostate cancer, whereas with decreased risks of breast and total cancer. The associations seen in the genetic studies are not necessarily causal, and their public health significance remains uncertain^691^. There is strong evidence that low folate status promotes cancer, especially colorectal cancer. However, evidence demonstrating a dose-response relationship between folate status and/or folate/folic acid intake within the normal human exposure ranges and increased rates of tumour growth in vivo is lacking. In general, there is no clear evidence from randomized controlled trials that supplementation/fortification with folic acid increases the cancer risk^1034,1035^. Most recent observational studies from 2021 revealed that the introduction of mandatory folic acid fortification of bread flour has not adversely affected colorectal cancer incidence in Australia^1180^ and that there was no evidence that high folate intake, both total and from synthetic forms, in the post-fortification period was related to increased colorectal cancer risk in this US female population^1181^. The latest meta-analysis of 24 cohort studies, mostly from the USA and Europe, involving 37,280 patients and 6,165,894 individuals has shown that high folate intake may be protective against colon cancer^1182^.

As regards folic acid maternal supplementation and health outcomes in children, there is no or limited evidence that children are at increased risk of atopy, asthma, wheezing, eczema, susceptibility to respiratory infection, childhood cancer, and autism spectrum disorders^246,671,691,1035,1173,1183–1186^.

In respect of unmetabolized folic acid in circulation, it has been pointed out that it is unlikely to be a new phenomenon. It is known that oral intake of folic acid above a certain threshold level (around 200 μg) results in an appearance of unmetabolized folic acid in the blood due to saturation of dihydrofolate reductase capacity. A number of studies conducted in countries with either mandatory or voluntary folic acid food fortification have reported detectable amounts of unmetabolised folic acid in the circulation in considerable proportions of adults and children. Dietary supplement use has increased in the U.S.A., while pregnant women have been prescribed folic acid tablets for nearly half a century, suggesting millions of person-years exposure to unmetabolized folic acid. The appearance of unmetabolized folic acid with a dose of 200 µg suggests that prior to fortification, any user of folic acid supplements would already have measurable unmetabolized folic acid, and any potential adverse effects would have been experienced. Biological and health consequences, if any, of unmetabolized folic acid are not established. Currently, there is no consistent evidence of adverse health effects causatively associated with circulating unmetabolized folic acid^128,671,680,691,944,982,988,995,1031,1035,1060,1133,1134,1139,1187–1189^.

Besides folic acid, 5-methyl-THF has also been allowed for food fortification in the European Union and other countries^626,627,1190–1192^. It is an important research question whether or not 5-methyl-THF is an effective and safer alternative to folic acid in providing supplemental levels of folate^190,197,235,236,674,680,1015,1190,1193^. Evidence for the efficacy of 5-methyl-THF in preventing neural tube defects is lacking at present^190,192,197,680,691,1152^. The utility of 5-methyl-THF is limited because it is less stable than folic acid in foods that undergo thermal processing^211,1022^. Using 5-methyl-THF may be advantageous for individuals with defects in the methylenetetrahydrofolate reductase enzyme who could have difficulty metabolizing folic acid from supplements or fortified foods to 5-methyl-THF by going straight to the next step in the metabolic pathway of vitamin B_9_^192,580,680,1034,1081,1152,1193–1196^. Use of 5-methyl-THF prevents the occurrence of unmetabolized folic acid in the peripheral circulation^192^.

Overall, regarding folic acid fortification, there is an inherent degree of uncertainty in nearly any aspect of scientific research. That is pertinent to the complex biological role of folate and its potential for both beneficial and adverse health effects depending on the dose and timing of exposure. The nature of science is that it cannot prove a negative. That is, there is no experimental design or methodology that can prove with 100% certainty that folic acid is completely ‘safe’^691^. Although the risk-benefit debate surrounding food fortification with folic acid continues among policymakers as well as researchers, the balance of available scientific evidence at this time indicates that the proven benefits of mandatory folic acid fortification outweigh the potential risks^128,651,679,687,691,1022,1034^. There are no established risks for adverse consequences resulting from existing mandatory folic acid fortification programs that have been implemented in many countries. Current folic acid fortification programs have been shown to support public health in populations^1035^. The effectiveness and safety of folic acid fortification programs have withstood the test of time^671,680^. There is also an interesting integrated risk-benefit analysis suggesting that a modest fortification with vitamin B_9_ will be very likely safe and suitable for prevention^1197^. However, additional research is needed to assess the health effects of folic acid supplement use, given the occurrence of some individuals and population groups exceeding the current tolerable upper intake level for folic acid. It is critical to evaluate all evidence for each of the concerns about potential harmful effects and to determine if there is a causal relation^680,1022,1031,1035^. Continued, careful, and effective monitoring should remain a key aspect of policy in this area, both to ensure that the target folic acid levels for beneficial effects are reached and to avoid any risk of overexposure in the population and potentially at-risk groups^128,614,1034,1066,1073^.

## Antifolate drugs

Folate metabolic reactions are essential for the proper function of all living cells but are especially critical for rapidly growing and dividing cells. Inhibition of folate-mediated biochemical reactions has been therefore successfully used in the therapy of pathological states involving such cells, where the predominant effect of antifolate medication facilitates a selective inhibitory effect: cancer, bacterial, and protozoal infections.

**Fig. 4:**
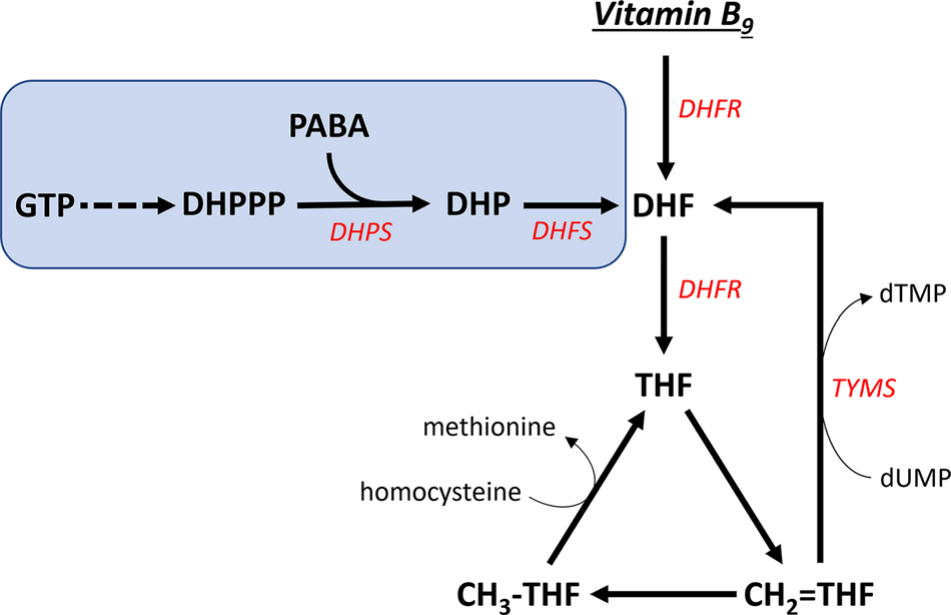
Antifolate drug targets. Summarized pathways of folate metabolism. The blue area marks de novo folate synthesis exclusive to bacteria and some protozoa. Principal enzymes targeted by antifolate drugs are highlighted in red. Protozoa express a bifunctional enzyme with dihydrofolate reductase and thymidylate synthase activity on a single protein. GTP guanosine triphosphate, DHPPP 6-hydroxymethyl-7,8-dihydropterin pyrophosphate, DHP 7,8-dihydropteroate, DHF dihydrofolate, THF tetrahydrofolate, CH_3_-THF 5-methyl-THF, CH_2_=THF 5,10-methylene-THF, DHPS dihydropteroate synthase, DHFS dihydrofolate synthase, DHFR dihydrofolate reductase, TYMS thymidylate synthase.

The mechanism of antifolate drugs differs depending on the target enzyme. Even if folate biochemistry comprises numerous enzymes, several of them have specific and crucial roles in the folate cycles which makes them relevant therapeutical targets (Fig. 4).

### Dihydrofolate reductase inhibitors

Dihydrofolate reductase is one of the most known and studied enzymes in folate metabolism. It has an important function in the THF regeneration from the thymidylate cycle and in THF production from dietary folate molecules. There are several differences between human, bacterial, and protozoal dihydrofolate reductase and folate biosynthetic pathways. A) Bacteria and some protozoa possess and use an endogenous folate biosynthetic pathway; however, certain parasitic protozoa like *Plasmodium sp*. and *Cryptosporidium sp*. have also salvage pathway that allows them to use exogenous folates^1198,1199^; while humans do not have the ability to synthesize folates de novo; B) Human and bacterial dihydrofolate reductase share high sequence homology, but structural differences are present that allow drugs selectively target the bacterial enzyme^1200^; C) Protozoa (e.g., *Plasmodium, Toxoplasma, Trypanosoma, Leishmania sp*.) have a bifunctional enzyme called dihydrofolate reductase-thymidylate synthase (DHFR-TYMS) in which dihydrofolate reductase and thymidylate synthase are two domains of a single homodimeric protein; in humans and bacteria, dihydrofolate reductase and thymidylate synthase occur as two separate, monofunctional proteins^1201^.

Antifolate drugs that act as dihydrofolate reductase inhibitors have been in therapeutical use for decades; the general overview is provided in Table 3. Novel candidate drugs were developed that inhibit not only dihydrofolate reductase but also thymidylate synthase and other enzymes in the thymidylate or purine cycles (AICAR formyl transferase, GARFT). Several drugs of this class have been investigated to their clinical effectiveness and safety. From the structural point of view, the “classical antifolates” are analogues of folate with pterin moiety: methotrexate, raltitrexed, pralatrexate, and pemetrexed. They do not passively cross the plasma membrane but use reduced folate carrier transporter to enter cells^1202,1203^, and they possess and require (poly)glutamate tail to utilize this active transport mechanism^1204^. “Non-classical” antifolates (piritrexim, trimetrexate, talotrexin, and nolatrexed) are lipophilic molecules that passively diffuse across cell membranes and do not require a specific transport mechanism. However, clinical studies showed satisfactory profiles for only a few candidate molecules that were approved for therapeutical use.

**Table 3:** Antifolate drugs

Drugs	Target enzyme	Indications
Methotrexate, pralatrexate, **(piritrexim), (talotrexin)**	Human dihydrofolate reductase	Cancer autoimmune diseases
Raltitrexed**, plevitrexed, (nolatrexed)**	Human dihydrofolate reductase thymidylate synthase GARFT	Cancer
Pemetrexed	Human dihydrofolate reductase thymidylate synthase GARFT AICAR formyl transferase	Cancer
Pyrimethamine, proguanil	Protozoal dihydrofolate reductase	Malaria, toxoplasmosis
Trimethoprim, brodimoprim, **(iclaprim)**	Bacterial dihydrofolate reductase	Antibiotic
Sulfonamides, dapsone	Bacterial DHFR	Antibiotic

FDA/EMA approved drugs are in bold; drugs in brackets have been withdrawn from clinical trials^1278^.

### Dihydropteroate synthase

In bacteria and some protozoa, dihydropteroate synthase is the first step in de novo synthesis of THF. Dihydropteroate synthase catalyses the production of 7,8-dihydropteroate from 6-hydroxymethyl-7,8-dihydropterin pyrophosphate (DHPPP) and *p*-aminobenzoic acid. Subsequently, dihydrofolate synthase adds glutamate to 7,8-dihydropteroate (DHP) and produces dihydrofolate, which enters the folate cycle when reduced to tetrahydrofolate via dihydrofolate reductase. Antifolate drugs that act as dihydropteroate synthase inhibitors cause a selective, very pronounced reduction in folate levels.

## Current clinical research on vitamin B_9_

In addition to above-mentioned issues, folic acid and derived drugs have been investigated in other pathological states both as possible drugs/preventive agents and as diagnostic tools.

One of the currently very intensively investigated targets is the folate receptor α. Pafolacianine, a modified folic acid conjugate with indocyanine green dye, binds to this receptor, which is prominently expressed in some cancers, and enables intraoperative tumour tissue imaging without serious adverse reactions^1205^. The same folate receptor is the target of the novel anticancer drug mirvetuximab soravtansine^1206–1208^; also another conjugate antibody farletuzumab with eribulin has been tested^1209^. There are also attempts to use the receptor as a base for vaccination^1210^ in cancer immunotherapy as well as to employ IgE antibodies against this receptor^1211^ for cancer treatment.

There are some recent clinical trials on the effect of folic acid or its close derivatives: Oral folinic acid supplementation might be beneficial in children with autism spectrum disorder^1212–1214^. The addition of folic acid increased the hypotensive effect of amlodipine^1215^. Similarly, patients suffering from hyperhomocysteinemia and hypertension administered with different doses of folic acid according to the genotype had a more pronounced decrease in arterial blood pressure when treated with another calcium channel blocker levamlodipine^1216^. Folic acid administration improved sexual function in postmenopausal women^1217^. A combination of folic acid with vitamin B_12_ could improve cognitive impairment in patients with Alzheimer´s disease^1218^ while the same combination had in general no effect on cognitive conditions in children aged 6–9 years^1219^. Interestingly, the effect of folic acid on cognition might be dependent on plasma levels of ω-3 fatty acids^1220^. In fact, folic acid combined with docosahexaenoic acid had a better effect on cognition than both compounds given in monotherapies^1221^.

The combination of vitamins B_9_ and B_12_ did not modify fracture risk^1222^. The combination of folic acid with vitamin B_12_ improved treatment outcomes in patients with type 2 diabetes mellitus, but the effect seems to be driven mostly by vitamin B_12_^1223^. Treatment with folic acid and zinc neither improved semen quality and live birth rates in couples seeking infertility treatment^1224^ nor modified sperm DNA methylation pattern^1225^. In patients with methylenetetrahydrofolate reductase gene 677 TT genotype, folic acid however improved seminal parameters^1226^.

## Conclusion

Vitamin B_9_ exists in several forms which are differently present in nature, have different stability, and different physiological functions. As this vitamin is crucial for humans and humans are not able to synthesize it, it must be taken from the diet or food supplements. Various ways of food preparation and storage impact in different ways its stability. There are several situations when a lack of this vitamin can be encountered. Its deficiency can lead to several severe consequences, including foetal malformation. For this reason, sufficient intake from food should be assured. The politics of different countries are not uniform as several countries have obligatory folate fortification while others are reluctant to this approach. Regardless, it seems that its fortification surpasses the claimed risks associated with a high intake of folates. Last but not least, given the differences in use and synthesis between humans and pathogens, healthy and tumour cells, there are several clinically used drugs targeting folate-dependent pathways.

## Supplementary information

Supplementary Information
