# Supplementary material for: Biological, dietetic and pharmacological properties of vitamin B9
Source: NPJ Sci Food. 2025 Mar 13;9:30. doi: 10.1038/s41538-025-00396-w (PMC11904035; doi:10.1038/s41538-025-00396-w)
Supplement: Supplementary file 1 — Supplementary Information [file 41538_2025_396_MOESM1_ESM.pdf]

Biological, dietetic and pharmacological properties of vitamin B_9

Tomáš Siatka, Marek Máťuš, Monika Moravcová, Patrícia Harčárová, Zuzana Lomozová, Kateřina Matoušová, Chaweewan Suwanvecho, Lenka Kujovská Krčmová & Přemysl Mladěnka*

Supplementary file

Table S1. Analytical techniques for detection of vitamin B_9 – details.

technique	sensitivity nmol/L	analytes	matrix	advantages	disadvantages	ref.	publication year
**LC-MS**	LLOQ<br>B_9-p-ABGA 3.0<br>B_9 5.0<br>B_9-MTHF 6.0	B_9, B_9-p-ABGA, B_9-MTHF including $B_{1,2,3,5,6,7,9}$, and its vitamers (21 analytes)	* plasma	* simple sample preparation<br>* MRM<br>* small sample volume (200 µL)	* complicated gradient elution	[1]	2015
	LLOQ<br>B_9 20 – 30	B_9, B_9-MTHF including $B_{6,12}$, and its	* mice plasma	* small sample volume (30 µL plasma, 20 mg	* complicated gradient elution	[2]	2015

	B_9-MTHF 20 – 30	metabolites (9 analytes)	* mice milk<br>* mice brain	milk or brain tissue)<br>* short analysis time<br>* various matrices			
	LLOQ<br>B_9-p-ABGA 0.03<br>B9 0.1<br>B_9-MTHF 0.07	B_9, B_9-p-ABGA, B_9-MTHF including $B_{1,2,3,6,}$ and its vitamers (18 analytes)	* breast milk	* small sample volume (200 μL)<br>* single sample preparation for 18 B-vitamers<br>* simple sample preparation	* complicated gradient elution<br>* complicated sample preparation of breast milk	[3]	2017
	LOQ<br>B_9 1.63×10^3	B_9 including $B_{1,2,3,5}$, B_6-PM, and fat soluble vitamins (12 analytes)	* serum<br>* tears	* one extraction for both groups of vitamins<br>* simple sample preparation<br>* MRM<br>* small sample volume (200 μL	* not sufficient validation parameters for all analytes	[4]	2018

				serum, 70 μL tears)			
LC-MS	LLOQ<br>B_9 29.84	B_9	* plasma	* simple sample preparation (protein precipitation)<br>* short run time (3.5 min)<br>* high throughput method<br>* MRM	* not possible to analyze metabolized B_9	[5]	2018
	LLOQ<br>B_9 0.2<br>B_9-MTHF 3.9	B_9, B_9-MTHF including $B_{2,6,}$ and its vitamers (11 analytes)	* plasma	* simple sample preparation (protein precipitation)<br>* small sample volume (50 μL)<br>* short run time (4 min)	* complicated step gradient profile	[6]	2018

	LOQ<br>B_9-FTHF 1.056<br>B_9 2.266	B_9 and its metabolites (7 analytes)	* whole blood<br>* mouse plasma<br>* mouse tissues	* rapid<br>* sensitive<br>* short runtime (5 min)<br>* single isotope labelled internal standard used	* not possible to estimate absolute value of folates metabolites	[7]	2018
	LOQ<br>B_9 0.39<br>B_9-THF 1.12 × 10^3	B_9, B_9-THF and including $B_{1,2,3,5,6,7,12,}$ and its vitamers (12 analytes)	* whole blood	* simple extraction	* validated only for 9 analytes<br>* SIM	[8]	2021
	LLOQ<br>B_9 0.6<br>B_9-MTHF 0.9	B_9, B_9-MTHF including $B_{2,5,}$ and B_6-PA	* serum	* automation of multicycle MSPE<br>* minimal systematic error<br>* sensitive<br>* cost effective	* need skilled operator for automatic instrument	[9]	2024

				* small sample volume (200 µL)			
	LOQ<br>B_9 407.79	$B_{2,9}$	* urine	* rapid extraction and detection (10 min)<br>* simple, and solvent saving sample preparation	* synthesis of nanocomposite<br>* 1 mL sample used	10	2021
**HPLC-PDA**	LOD<br>B_9-MTHF 0.39<br>B_9 0.48	B_9, B_9-MTHF including B_{12}	* serum	* simple sample preparation<br>* rapid<br>* cheap<br>* small sample volume (200 µL)<br>* low reagents consumption	* not sufficient validation parameters for all analytes	11	2022

**Sensors / nanodots / CL / FLD / ECD**	LOD<br>B_9 0.28	B_9	* serum	* selective<br>* no need of pH adjustment in sample preparation	* electrode prepared in laboratory<br>* not available in the market	[12]	2017
	LOD<br>B_9 0.5	B_9	* serum	* sensitive<br>* easy to synthesize coated nanoparticles one-step chemical reaction<br>* convenient and better separation from sample with magnetic solid phase extraction	* synthesis of carbon quantum dots<br>* synthesis of magnetic nanoparticles<br>* 1 mL serum required	[13]	2017
	LOD<br>B_9 36.8	B_9	* serum	* no need of organic solvents	* not available in the market	[14]	2018

	LOD<br>B_9 1 × 10^3	B_9	* urine	* good stability<br>* selective	* Synthesis of graphene foam<br>* not available in the market<br>* poor sensitivity	15	2018
	LOD<br>B_9 4.58	B_9	* urine	* simple sample preparation<br>* selective	* not available in the market	16	2019
**Sensors / nanodots / CL / FLD / ECD**	LOD<br>B_9 4	B_9	* serum<br>* urine	* simple sample preparation	* not available in the market	17	2019
	LOD<br>B_9 37	B_9	* serum	* fast<br>* easy<br>* economical and eco-friendly sensor preparation process<br>* selective	* synthesis of nanocomposit e<br>* electrode prepared in laboratory	18	2020

				* no need pH adjustment of sample			
	LOD B_9 49	B_9	* serum<br>* plasma	* sensitive<br>* sensor with high fluorescent yield and good chemical stability	* sensor prepared in laboratory	[19]	2020
	LOD B_9 300	B_9	* serum	* visual fluorescent probe<br>* selective<br>* No need expensive and complex instrumentation	* synthesis of quantum dots<br>* fluorescent probe prepared in laboratory	[20]	2021
	LOD B_9 0.47	B_9	* serum<br>* urine	* no need of organic solvents in sample preparation<br>* one-pot synthesis	* synthesis of nanoclusters	[21]	2021

				* high storage stability			
	LOD<br>B_9 0.72	B_9	* serum	* rapid detection response<br>* high stability<br>* robust sensor matrix	* synthesis of nanosheet and nanocomposit e<br>* sensor prepared in laboratory	[22]	2022
	LOD<br>B_9 30	B_9	* serum	* sensitive<br>* storage stable of probe<br>* one-step reaction of nanocomposite synthesis	* synthesis of nanocomposit e<br>* sensor prepared in laboratory	[23]	2022
**Sensors / nanodots / CL / FLD / ECD**	LOD<br>B_9 142	B_9	* serum	* simple sample preparation and synthesis<br>* no impact from serum matrix	* synthesis of fluorescent probe	[24]	2023

				* sensitive			
	B_9 LOD 0.63	B_9	* serum (pregnant women)<br>* artificial cerebrospinal fluid	* simple sample preparation<br>* small volume sample (not specified in article)	* electrode prepared in laboratory<br>* need period at low concentration<br>* higher LOD than ELISA kit	[25]	2023
	LOD B_9 470	B_9	* serum	* simple sample preparation (dilution)<br>* selective	* synthesis of nanocluster<br>* sensor prepared in laboratory	[26]	2024
**Microbiological test kits**	LLOQ B_9 6.8	B_9	* serum	* small sample volume (100 μL)	* high price (working in duplicate recommended)<br>* long analysis time (24 h)	[27]	2024

**ELISA kit**	LOD<br>B_9 62.08 × 10^{-3}	B_9	* serum<br>* plasma<br>* cell culture supernatant<br>* tissue	* small sample volume (250 μL)<br>* one kit for various matrices<br>* sensitive	* for research only<br>* cross reactivity with analogues<br>* time and money consuming for small sample series	[28]	2021
	LOD<br>B_9 0.04	B_9	* serum<br>* plasma<br>* tissue homogenate<br>* cell lysates<br>* cell culture supernatant<br>* others	* small sample volume (50 μL)<br>* one kit for various matrices	* for research only<br>* time and money consuming for small sample series	[29]	2024

	LOD<br>B_9 0.05	B_9	* serum<br>* plasma<br>* cell<br>* tissue	* small sample volume (50 µL)<br>* one kit for various matrices	* for research only<br>* time and money consuming for small sample series	30	2024

LOD Limit of Detection, LOQ Limit of Quantification, LLOQ Lower Limit of Quantification

B_1 Thiamine; B_2 riboflavin; B_3 Niacinamide; B_5 pantothenic acid; B_6 pyridoxine; B_6-PM pyridoxamine; B_6-PA pyridoxic acid; B_7 biotin; B_9 folic acid; B_9-p-ABGA para-aminobenzoyl glutamic acid; B_9-THF tetrahydrofolic acid; B_9-FTHF 5-formyltetrahydrofolate; B_9-MTHF 5-methyltetrahydrofolate; B_{12} cyanocobalamin

CL Chemiluminescence; ECD Electrochemical Detection; FLD Fluorescence Detection; LC-MS Coupling of Liquid Chromatography and Mass Spectrometry; MRM Multiple Reaction Monitoring; SIM Selected Ion Monitoring;

References:

1. Redeuil, K. M. et al. Simultaneous quantification of 21 water soluble vitamin circulating forms in human plasma by liquid chromatography-mass spectrometry. *J. Chromatogr. A* **1422,** 89-98; 10.1016/j.chroma.2015.09.049 (2015).
2. Oosterink, J. E. et al. Accurate measurement of the essential micronutrients methionine, homocysteine, vitamins B6, B12, B9 and their metabolites in plasma, brain and maternal milk of mice using LC/MS ion trap analysis. *J. Chromatogr. B* **998-999,** 106-113; 10.1016/j.jchromb.2015.07.008 (2015).

3. Redeuil, K. et al. A novel methodology for the quantification of B-vitamers in breast milk. *J. Anal. Bioanal. Tech.* **8,** 352; 10.4172/2155-9872.1000352 (2017).
4. Khaksari, M. et al. Detection and quantification of vitamins in microliter volumes of biological samples by LC-MS for clinical screening. *AIChE J.* **64,** 3709-3718; 10.1002/aic.16345 (2018).
5. Zayed, A., Bustami, R., Alabsi, W. & El-Elimat, T. Development and validation of a rapid high-performance liquid chromatography–tandem mass spectrometric method for determination of folic acid in human plasma. *Pharmaceuticals* **11,** 52; 10.3390/ph11020052 (2018).
6. Asante, I. et al. Simultaneous quantitation of folates, flavins and B6 metabolites in human plasma by LC–MS/MS assay: applications in colorectal cancer. *J. Pharm. Biomed. Anal.* **158,** 66-73; 10.1016/j.jpba.2018.05.030 (2018).
7. Nandania, J., Kokkonen, M., Euro, L. & Velagapudi, V. Simultaneous measurement of folate cycle intermediates in different biological matrices using liquid chromatography–tandem mass spectrometry. *J. Chromatogr. B* **1092,** 168-178; 10.1016/j.jchromb.2018.06.008 (2018).
8. Kahoun, D. et al. Development and validation of an LC-MS/MS method for determination of B vitamins and some its derivatives in whole blood. *PLOS One* **17,** e0271444; 10.1371/journal.pone.0271444 (2022).
9. Gu, Y. et al. A novel automated multi-cycle magnetic solid-phase extraction coupled to LC-MS/MS to study the disorders of six functional B vitamins in patients with gastroenterology and hyperhomocysteinemia. *J. Pharm. Biomed. Anal.* **241,** 115989; 10.1016/j.jpba.2024.115989 (2024).
10. Kang, L. et al. Rapid determination of folic acid and riboflavin in urine by polypyrrole magnetic solid-phase extractant combined ultra-performance liquid chromatography. *J. Chromatogr. A* **1648,** 462192; 10.1016/j.chroma.2021.462192 (2021).
11. Akbari, A. et al. Determination of B Vitamins by Double-Vortex-Ultrasonic Assisted Dispersive Liquid–Liquid Microextraction and Evaluation of their Possible Roles in Susceptibility to COVID− 19 Infection: Hybrid Box–Behnken Design and Genetic Algorithm. *J. Chromatogr. Sci.* **60,** 897-906; 10.1093/chromsci/bmab124 (2022).

12. Xu, H. et al. Voltammetric determination of folic acid at physiological pH values by using a glassy carbon electrode modified with a multilayer composite consisting of polyoxometalate (H 8 P 2 Mo 16 V 2 O 62) and reduced graphene oxide and prepared via layer-by-layer self-assembly and in-situ photoreduction. *Mikrochim. Acta* **184,** 4295-4303; 10.1007/s00604-017-2447-1 (2017).
13. Wang, M. et al. Nitrogen-doped carbon quantum dots as a fluorescence probe combined with magnetic solid-phase extraction purification for analysis of folic acid in human serum. *Anal. Bioanal. Chem.* **409,** 7063-7075; 10.1007/s00216-017-0665-3 (2017).
14. Kıranşan, K. D. &Topçu, E. Free-standing and flexible MoS2/rGO paper electrode for amperometric detection of folic acid. *Electroanalysis* **30,** 810-818; 10.1002/elan.201700778 (2018).
15. Gao, X. et al. Synthesis of graphene/ZnO nanowire arrays/graphene foam and its application for determination of folic acid. *J. Electroanal. Chem.* **808,** 189-194; 10.1016/j.jelechem.2017.12.017 (2018).
16. Güney, S. Electrochemical synthesis of molecularly imprinted poly (p-aminobenzene sulphonic acid) on carbon nanodots coated pencil graphite electrode for selective determination of folic acid. *J. Electroanal. Chem.* **854,** 113518; 10.1016/j.jelechem.2019.113518 (2019).
17. Hussain, S. et al. Facile preparation of molybdenum carbide (Mo2C) nanoparticles and its effective utilization in electrochemical sensing of folic acid via imprinting. *Biosens. Bioelectron.* **140,** 111330; 10.1016/j.bios.2019.111330 (2019).
18. Wang, Q. et al. A fast and facile electrochemical method for the simultaneous detection of epinephrine, uric acid and folic acid based on ZrO2/ZnO nanocomposites as sensing material. *Anal. Chim. Acta* **1104,** 69-77; 10.1016/j.aca.2020.01.012 (2020).
19. Yang, B. et al. A water-stable MOF-AgClO4-abtz as fluorescent sensor for detection of folic acid based on inner filter effect. *Talanta* **217,** 121019; 10.1016/j.talanta.2020.121019 (2020).
20. Yang, M. et al. Visual detection of folic acid based on silica coated CdTeS quantum dots in serum samples. *Mater. Res. Bull.* **144,** 111509; 10.1016/j.materresbull.2021.111509 (2021).
21. Fereja, S. L. et al. Silver-enhanced fluorescence of bimetallic Au/Ag nanoclusters as ultrasensitive sensing probe for the detection of folic acid. *Talanta* **233,** 122469; 10.1016/j.talanta.2021.122469 (2021).
22. Yadav, D. et al. Nanohybrid Comprising Gold Nanoparticles–MoS2 Nanosheets for Electrochemical Sensing of Folic Acid in Serum Samples. *Electroanalysis* **35,** e202200286; 10.1002/elan.202200286 (2023).

23. Sun, Y., Wang, X. & Zhang, H. Sensitive and Stable Electrochemical Sensor for Folic Acid Determination Using a ZIF-67/AgNWs Nanocomposite. *Biosensors* **12,** 382; 10.3390/bios12060382 (2022).
24. Li, K., Quan, X. & Yan, B. Eu (III)-functionalized iCOF hybrids by “tandem post-synthetic modifications” for fluorescent detection of folic acid and trimethoprim: a logical judgement by combination of neural networks and logic gates. *Sens. Actuators B Chem.* 134078; 10.1016/j.snb.2023.134078 (2023).
25. Xu, Y. et al. In situ electrodeposition of bismuth oxide nanowires@ MWNT on the carbon fiber microelectrode for the sensitively electrochemical detection of folic acid. *Talanta* **253,** 123944; 10.1016/j.talanta.2022.123944 (2023).
26. Vegad, Y. et al. Folic acid detection using β-cyclodextrin-functionalized copper nanoclusters and vitamin B6 cofactor pyridoxal. *ACS Appl. Nano Mater.*; 10.1021/acsanm.3c05697 (2024).
27. Immundiagnostik, A. G. ID-Vit® Folic acid, https://www.immundiagnostik.com/media/pages/testkits/kif005/880242fdc4-1679409720/kif005_2022-07-05_folsaeure.pdf
28. LSBio All species Folic Acid ELISA Kit (Competitive EIA), https://www.lsbio.com/elisakits/manualpdf/ls-f4330.pdf
29. abbexa Folic Acid / Vitamin B9 ELISA Kit, https://www.abbexa.com/documents/manual/abx150387_ifu.pdf (2023).
30. Cell Biolabs, I. Folic Acid ELISA Kit, https://www.cellbiolabs.com/sites/default/files/MET-5068-folic-acid-elisa-kit.pdf
